# Current Prognostic Biomarkers for Peripheral Arterial Disease: A Comprehensive Systematic Review of the Literature

**DOI:** 10.3390/metabo15040224

**Published:** 2025-03-25

**Authors:** Hamzah Khan, Natasha R. Girdharry, Sophia Z. Massin, Mohamed Abu-Raisi, Gustavo Saposnik, Muhammad Mamdani, Mohammad Qadura

**Affiliations:** 1 Division of Vascular Surgery, St. Michael’s Hospital, Toronto, ON M5B 1W8, Canada; hamzah.khan@mail.utoronto.ca (H.K.);; 2 Li Ka Shing Knowledge Institute, St. Michael’s Hospital—Unity Health Toronto, Toronto, ON M5B 1T8, Canada; 3 Mount Sinai Hospital, Toronto, ON M5G 1X5, Canada; 4 Toronto General Hospital, University Health Network, Toronto, ON M5G 2C4, Canada; 5 Division of Neurology, Department of Medicine, University of Toronto, Toronto, ON M5S 3H2, Canada; 6 Vascular Surgery, Heart, Vascular & Thoracic Institute, Cleveland Clinic, Abu Dhabi PO Box 112412, United Arab Emirates

**Keywords:** peripheral artery disease, vascular, biomarkers, plasma, protein, prognosis

## Abstract

**Background:** Peripheral arterial disease (PAD) is a chronic atherosclerotic disease characterized by atheromatous plaque buildup within arteries of the lower limbs. It can lead to claudication, skin ulcerations, and, in severe cases, chronic limb-threatening ischemia, requiring amputation. There are several plasma protein biomarkers that have been suggested as prognostic markers for adverse events, including major adverse cardiovascular and limb events. However, the clinical benefit and ability to clinically adapt these biomarkers remains uncertain due to inconsistent findings possibly related to heterogenous study designs and differences in methodology. **Objectives:** This review aims to evaluate the current literature on the prognostic value of plasma protein biomarkers for PAD, their predictive ability for PAD-related adverse outcomes, and their potential roles in guiding PAD management. **Methods:** To address these challenges, we conducted a systematic review of MEDLINE, Embase, and Cochrane CENTRAL libraries of the current literature (2010–2024). **Results:** We found 55 studies that evaluated the prognostic value of 44 distinct plasma proteins across various pathophysiological processes. These included markers of immunity and inflammation, markers of metabolism, cardiac biomarkers, markers of kidney function, growth factors and hormones, markers of coagulation and platelet function, extracellular matrix and tissue remodeling proteins, and transport proteins. This review summarizes the existing evidence for prognostic protein plasma biomarkers for PAD and their association with adverse events related to PAD. Conclusions: With this review, we hope to provide a comprehensive list of the prognostic markers and their value as prognostic biomarkers to guide clinical decision making in these patients.

## 1. Introduction

Peripheral arterial disease (PAD) is a devastating cardiovascular disease that affects over 200 million individuals worldwide [1]. It is characterized by atherosclerotic plaque buildup within the arterial wall layer of the peripheral arteries, most commonly within the lower limbs, leading to a reduction in blood flow to the affected areas [2,3]. Consequently, patients are faced with debilitating claudication and, in severe cases, require surgical interventions or amputation with a mortality of over 50% post amputation [4,5,6]. Despite its vast prevalence of over 1.5% of the global population, PAD remains underdiagnosed and sub-optimally managed, putting this patient population at increased risk of life-altering adverse cardiovascular events, such as myocardial infarctions (MIs), cerebrovascular attacks (CVAs), major limb amputations, and death [7,8]. Subsequently, PAD patients are reported to have worse outcomes when compared to those with coronary artery disease (CAD), despite both being atherosclerotic in nature [9]. Unlike CAD, for which Troponin is a strong clinical biomarker, a PAD-specific biomarker that facilitates quick screening assessment and tools determining the prognosis and clinical course of PAD patients are severely lacking. The Society for Vascular Surgery (SVS) guidelines recommend an individualized approach considering other clinical factors beyond arterial anatomy, such as postprocedural risk, availability of conduit, and anticipated risk of wound complications [10]. There is currently a lack of biomarkers available that are clinically relevant with widespread use for the prognosis of PAD. The purpose of this review is to evaluate the current literature on the prognostic value of plasma protein biomarkers for PAD assess their predictive ability for PAD-related adverse outcomes and their potential roles in guiding PAD management. In this up-to-date and in-depth systematic review, all current research on prognostic plasma protein biomarkers for PAD was investigated and all proteins found within the review were categorized based on their primary physiological function. Each proteins prognostic capability was assessed and their associations with PAD-related events and their potential clinical utility were analyzed. Through this review, we hope to further elucidate the pathophysiology of PAD and allow for further research in the use of these markers as prognostic markers for PAD risk stratification and validation of these markers in a clinical setting.

## 2. Methodology

The study was conducted following the PRISMA Statement; however, this review was not registered with PROSPERO. MEDLINE, Embase, and Cochrane CENTRAL libraries were searched for original research articles, including prospective or retrospective cohort studies, case–control studies, or randomized controlled trials, published between January 2010 and May 2024. Our literature search was conducted using free-text terms without restricting to Medical Subject Headings (MeSH). The search terms included “Peripheral artery disease” cross-searched with “protein”, “plasma”, and “prognosis” to maximize sensitivity in retrieving relevant studies. Studies were further filtered for English and human research only. All studies investigated were chosen based on a predefined inclusion and exclusion criteria. Studies that were included were required to be investigating patients diagnosed with PAD and assessing the prognostic value of plasma protein biomarkers in prediction of cardiovascular or PAD-related adverse outcomes. This included disease progression, myocardial infarction, stroke, need for surgical intervention, amputation, and cardiovascular-related mortality. Exclusion criteria included non-human studies, research focusing on non-blood-based biomarkers, such as tissue, genetic, or cellular markers, and studies that solely examined diagnostic markers rather than prognostic outcomes. Additionally, studies that assessed biomarkers exclusively in the post-surgical period without a pre-surgical prognostic component were excluded. Case reports, editorials, reviews, letters to the editor, and conference abstracts without original data were also excluded. Abstracts and titles were screened for related research, followed by full-text review. Two independent reviewers screened titles and abstracts based on predefined inclusion and exclusion criteria. Full-text reviews were then conducted for eligible studies, and any discrepancies in study selection were resolved through discussion. Risk of bias assessment was not conducted on the analysis. Studies that investigated only diagnostic markers, non-protein markers, tissue markers, non-blood-based markers, or post-surgical markers were excluded. This review was conducted through Covidence (Covidence.org).

## 3. Results

**Figure 1:**
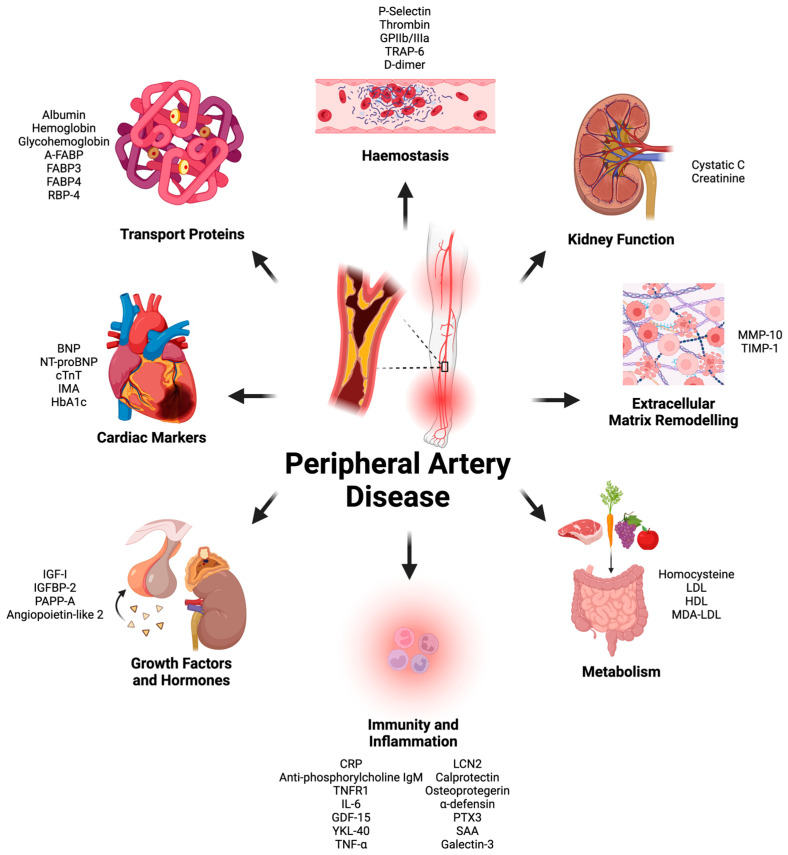
All protein plasma biomarkers found during literature review split based on primary physiological function. Glycoprotein (GP), thrombin receptor activator for peptide (TRAP), brain natriuretic peptide (BNP), N-terminal proBNP (NT-proBNP), matrix metalloproteinase (MMP), tissue inhibitors of metalloproteinases (TIMP), low-density lipoprotein (LDL), high-density lipoprotein (HDL), malondialdehyde-modified low-density lipoprotein (MDA/LDL), c-reactive protein (CRP), tumor necrosis factor receptor (TNFR), interleukin (IL), growth differentiation factor (GDF), Chitinase 3-like 1 (YKL-40), Serum amyloid A (SAA), tissue necrosis factor (TNF), Lipocalin-2 (LCN2), pentraxin (PTX), insulin like growth factor (IGF), insulin-like growth-factor-binding protein (IGFBP), pregnancy-associated plasma protein A (PAPP-A), cardiac muscle troponin T (cTnT), ischemia-modified albumin (IMA), hemoglobin A1c (HbA1c), ratty-acid-binding protein (FABP), adipose-FABP (A-FABP). This graph was created by Hamzah Khan using Biorender (biorender.com).

**Figure 2:**
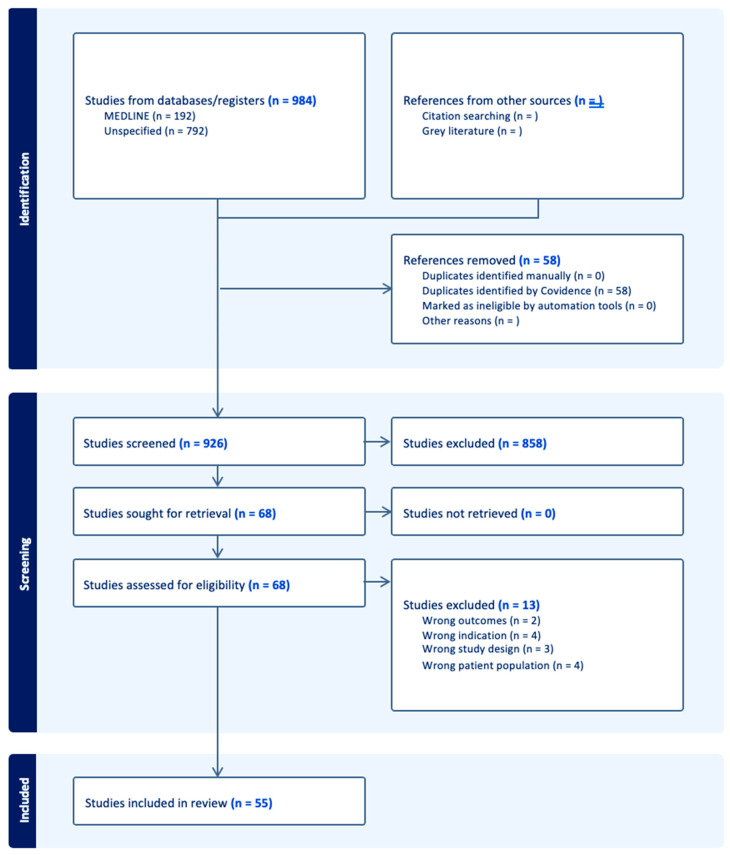
PRISMA diagram summary of systematic review articles included and excluded during literature search.

There were forty-four proteins determined as potential prognostic biomarkers for PAD in this literature review. Based on the primary physiological function of each protein, the proteins were categorized into eight distinct categories: markers of immunity and inflammation, markers of metabolism, cardiac biomarkers, markers of kidney function, growth factors and hormones, markers of coagulation and platelet function, extracellular matrix and tissue-remodeling proteins, and transport proteins (Figure 1). A total of 58 studies were included in this systematic review (Figure 2 and Table 1).

### 3.1. Markers of Immunity and Inflammation

PAD is being increasingly recognized as a chronic inflammatory disease, with inflammation potentially playing a key role in the processes associated with its initiation and progression. The inflammatory process occurs due to the progressive atherosclerotic plaque buildup within the arterial wall, leading to the recruitment and activation of immune cells [65]. These inflammatory processes can lead to further endothelial cell damage, plaque instability, arterial narrowing, and may contribute to adverse events, including progression of the disease and the risk of CLTI. Several inflammatory biomarkers have been studied as potential prognostic markers of PAD. Due to the importance of inflammation in the disease process, investigating these markers may be important for prognostication of PAD. In this review, 14 inflammatory proteins were found to be investigated for their prognostic capabilities in patients with PAD.

#### 3.1.1. C-Reactive Protein (CRP)

C-Reactive Protein (CRP) is an acute phase protein that is produced by the liver in response to inflammation. Its release can be triggered by many cytokines and leads to the immune destruction and clearance of pathogens, damaged cells, and foreign substances [66]. CRP has been well established as a prognostic marker for cardiovascular disease and has been incorporated into many diagnostic and prognostic risk assessment models [67]. In this review, CRP was the most common plasma biomarker for prognosis of PAD, with twenty-one studies including this protein in their study. Out of the twenty-one studies, eight studies demonstrated an association of high CRP with increased risk of major adverse cardiovascular events (MACEs), which included MI, CVA, and cardiovascular-related death with a wide range of demonstrated hazard ratios between 1.01 and 3.59 [13,22,26,28,31,55,57,58]. Interestingly, three studies demonstrated no association with MACE [14,25] or ischemic heart disease events and CRP levels [32]. Three studies investigated CRP plasma levels and their association with graft restenosis or failure, with one study demonstrating that at one-year post-endovascular therapy, higher CRP levels were associated with an increased risk of the need for reintervention (HR 1.1, 95% CI: 1.05–1.2, *p* < 0.001). Another study demonstrated a stronger association between higher CRP and graft failure (HR 2.11, 95% CI: 1.09–4.07, *p* = 0.03) [23], but Songlig et al. demonstrated no association between in-stent stenosis and baseline CRP [62]. Three studies agreed that increasing CRP at baseline leads to an increased risk of PAD and CLI [17,18,39]. Four studies investigated CRP levels and the risk of major adverse limb events (MALEs), which included the need for surgical intervention, minor and major limb amputation, and death. Of these studies, two demonstrated strong associations with CRP and MALE [30,50], with one demonstrating an association between CRP and non-fatal PAD events between the first and fourth quartile (HR 2.48, 95% CI: 1.85–3.32, *p* < 0.001) [29] and one demonstrating no association with CRP and MALE [33]. Though CRP has been used often for risk prediction, it has quite a wide range of uses as an inflammatory marker and may not be specific enough for outcomes related to PAD.

#### 3.1.2. Interleukin-6 (IL-6)

Interleukin-6 (IL-6) is a marker transiently produced by lymphocytes and monocytes in response to injury, pathogens, and activated host immunity [68]. Once released into the bloodstream, it can induce rapid release of other acute-phase proteins, such as CRP and fibrinogen [69]. In the bone marrow, IL-6 causes the maturation of megakaryocytes, inducing the production and release of platelets into the bloodstream [70]. Currently, IL-6 is used as a prognostic marker in several diseases, including cancer, cardiovascular disease, and lung disease [71,72,73]. In cardiovascular disease, due to its influence of inflammation, it has been demonstrated to increase the risk of MI, stroke, and cardiovascular death [73]. In this review, four studies investigated the prognostic capability of IL-6 in patients with PAD [23,33,50,62]. A study conducted by Biscetti et al. in 2019 found that increasing levels of IL-6 were associated linearly and independently with increased risk of MACEs and MALEs [50]. Conversely, Takamura et al. in 2017 had previously demonstrated no association with either clinical endpoint of MACE or MALE [33]. Guo et al. conducted a study in 88 patients with occlusive femoropopliteal artery disease undergoing stent implantation and determined the association between CRP levels and IL-6 and in-stent stenosis. They determined that elevated IL-6 was associated with increase odds of 6-month in-stent stenosis with an odds ratio of 1.11 (95% CI: 1.00–1.23, *p*  =  0.044) [62]. In a study in 2019, conversely, in a group of 142 patients with PAD undergoing infra-inguinal autogenous vein bypass, IL-6 was not associated with graft failure [23].

#### 3.1.3. Growth Differentiation Factor 15 (GDF15)

Growth differentiation factor 15 (GDF15) is a protein belonging to the transforming growth factor-beta (TGF-β) superfamily and involved with the regulation of the cellular stress response. It is a cytokine that rapidly increases in expression in response to tissue injury, hypoxia, oxidative stress, and inflammation [74]. It is hypothesized to prepare the body for stress through reduction in appetite, food intake, and body mass [75]. GDF15 has been established as a marker of cancer metastasis in pancreatic cancer, and its regulation has also been investigated as a possible treatment for obesity and diabetes [76,77]. Studies have demonstrated that due its association with inflammation, GDF15 may potentially act as a biomarker for cardiovascular disease. It has been demonstrated to be a marker of atherosclerosis, vascular stress by hypoxia, as well as associated with adverse cardiovascular outcomes in patients with coronary artery disease [78]. In this review, we found one study investigating the prognostic value of GDF15 in predicting adverse cardiovascular events in patients with PAD. The authors demonstrated that GDF-15 was associated with adverse cardiovascular outcomes within 5.2 years with a hazard ratio of 2.32 (95% CI: 1.70–3.17, *p* < 0.001) [24].

#### 3.1.4. Chitinase-3-Like Protein 1 (CHI3L1/YKL-40)

Chitinase-3-like protein 1 (CHI3L1), also known as YKL-40, is a protein belonging to the glycoside hydrolase family 18 family and is secreted by several cells, including macrophages, chondrocytes, and synovial cells. It is associated with the pathogenic processes of inflammation, fibrosis, and asthma [79]. It has been suggested to influence the activation of macrophages, cell proliferation, and angiogenesis [79]. A recent review demonstrated CHI3L1 could potentially be a predictor of cardiovascular disease, as it has been shown to be associated with vascular inflammation, and elevated levels are associated with cardiovascular disease progression [80]. One study was found through this review that investigated CHI3L1 in 86 patients with PAD and 612 healthy individuals and noted that elevated CHI3L1 was associated with increased risk of PAD (*p* = 3.3 × 10−23) [34].

#### 3.1.5. Serum Amyloid A (SAA)

Serum amyloid A (SAA) is an acute-phase protein produced by the liver in response to a wide variety of cytokines, including IL-6, IL-1β, and TNF-α [81]. It primarily functions by removing toxic lipids produced during cellular injury and inflammatory responses and redirects HDL and cholesterol during tissue repair [82]. Recent studies have demonstrated that elevated SAA may influence atherosclerotic plaque stability and lead to increased risk of adverse cardiovascular events [83]. In our review, one study investigated SAA’s association with ischemic heart disease events in 595 patients with PAD. They noted that SAA levels were not different between time periods leading up to ischemic heart disease events [32].

#### 3.1.6. Tumor Necrosis Factor-α (TNF-α)

Tumor necrosis factor-α (TNF-α) is a proinflammatory cytokine that is released by activated macrophages, T cells, and endothelial cells in response to infection, tissue injury, and inflammation [84]. After its release, it binds to its receptors TNFR1 and TNFR2, which triggers downstream signaling cascades, activating NF-κB and MAPK pathways and leading to further immune cell activation, inflammation, and apoptosis [84]. TNF-α is well known for its influence on cardiovascular disease by influencing endothelial dysfunction, atherosclerotic plaque formation, and vascular remodeling. Increasing levels of TNF-α have demonstrated increased risk of coronary artery disease (OR 2.25, 95% CI: 1.50–3.37) and ischemic stroke (OR 2.27, 95%CI: 1.50–3.43) [85]. In relation to PAD, one study investigated the prognostic capability of TNF-α [50]. In 2019, Biscetti et al. studied 299 patients with occlusive disease below the knee and demonstrated that TNF-α exhibited a significant correlation with the risk of both MALE and MACE at 12 months after baseline (*p* < 0.001) [50].

#### 3.1.7. Tumor Necrosis Factor-α Receptor 1 (TNFR1)

Tumor necrosis factor-α receptor 1 (TNFR1) is a membrane-bound receptor found on all human tissue that mediates the response of TNF-α. Its activation leads to different signal cascades, including triggering pathways such as NF-κB, MAPK, and caspase pathways, which regulate immune responses, inflammation, cell survival, and apoptosis [86,87]. TNFR1 has been previously associated with increased risk of loss of kidney function, as well as greater baseline disability and disability over time due to stroke [88,89]. In patients with PAD, two studies investigated the association between TNFR1 and PAD. In a study conducted in 2018, 1412 participants were followed for an average of 5.6 years. In this group, 112 developed lower-extremity arterial disease (LEAD). The researchers found that between the third and first tertiles of TNFR1, there was a significantly higher rate in the development of LEAD in the higher tertile (HR 2.16, 95% CI: 1.19–3.92, *p* = 0.01) [17]. In a second study conducted by the same group, 1395 participants were followed for 5.8 years, during which 66 required aortic or lower-limb revascularization. Similarly, they determined that between third and first tertiles, TNFR1 was significantly associated with the need for revascularization (HR 2.16, 95% CI: 1.19–3.92, *p* = 0.01) [18].

#### 3.1.8. Lipocalin-2 (LCN2)

Lipocalin-2 (LCN2), also known as neutrophil gelatinase-associated lipocalin (NGAL), is an adipocytokine responsible for carrying small hydrophobic molecules, such as cholesterol, free fatty acids, and hormones, to their target organs [90]. LCN2 also has functions within the innate immune system by sequestering iron to reduce availability for pathogens [91]. It has been demonstrated to have prognostic capabilities for kidney function and is expressed in acute kidney injury and release after tubular damage. It can also be found highly concentrated within atherosclerotic plaque, with increasing concentrations correlating with coronary artery disease severity [92]. In this review, one study was found that determined the relationship between LCN2 and cardiovascular-related death and amputation. They determined that LCN2 increased the risk of cardiovascular-related death and amputation by 5.6 folds (*p* < 0.001) [52].

#### 3.1.9. Calprotectin

Calprotectin is a calcium-binding protein that is important for inflammation and innate immunity, binding to essential metal ions, such as zinc and manganese, to prevent antimicrobial growth [93]. Calprotectin also acts as a damage-associated molecular pattern (DAMP)-signaling tissue injury and initiates inflammation and the immune response [94]. Studies have shown associations between calprotectin and cardiovascular disease, with every one-unit increase in calprotectin leading to a 1.26-fold (95% CI, 1.13–1.41) increase in risk of cardiovascular disease. It has also been associated with cardiovascular disease risks such as hypertension [95,96]. In regard to PAD, one study investigated the association between calprotectin and PAD and determined that calprotectin increased the risk of cardiovascular-related death or amputation by 1.8 folds (*p* = 0.034) [52].

#### 3.1.10. Osteoprotegerin (OPG)

Osteoprotegerin (OPG), also known as tumor necrosis factor receptor superfamily member 11B (TNFRSF11B), is a member of the tumor necrosis factor receptor superfamily and is associated with bone homeostasis. It functions through the inhibition of osteoclast formation and the prevention of bone resorption by acting as a decoy receptor to the receptor activator of nuclear factor kappa B ligand (RANKL) [97]. Its functions have been demonstrated to be beyond bone homeostasis, with functions in vascular biology, inflammation, and fibrosis. Specifically within the vasculature, it has been demonstrated to be a marker of vascular injury and inflammation [98] as well as a marker of progressive atherosclerosis and arterial calcification [99]. Due to its influence on fibrosis and vascular injury, one study investigated the use of OPG as a prognostic marker of PAD. The researchers followed 299 patients with occlusive disease below the knee and demonstrated that OPG exhibited a significant correlation with the risk of both MALE and MACE at 12 months after baseline (*p* < 0.001) [50].

#### 3.1.11. α-Defensin

α-Defensin is a small cationic peptide produced by neutrophils that acts to prevent viral and fungal infections by membrane disruption, pore formation, and by the inhibition of pathogen replication [100]. α-Defensin can also bind to Toll-like receptors (TLRs) and modulate the immune response through increasing or decreasing inflammatory signaling [101]. α-Defensin has been found within atherosclerotic lesions, and it is suggested that α-Defensin influences plaque stability by reducing LDL metabolism and promoting fibrinolysis [102]. A study in patients with coronary artery disease demonstrated the prognostic capability of α-Defensin, showing that it is an independent predictor of mortality and recurrent percutaneous coronary intervention in patients with stable CAD [103]. In patients with PAD, a study investigated 463 patients with lower-extremity peripheral arterial disease and determined that high levels of α-Defensin was associated with an increased risk of cardiovascular mortality (HR 3.04 95% CI 1.26–7.32; *p* = 0.013).

#### 3.1.12. Plasma Pentraxin 3 (PTX3)

Pentraxin 3 (PTX3) is a long pentraxin protein (compared to CRP, which is a small pentraxin) produced by a wide range of cellular types, including macrophages, dendritic cells, endothelial cells, and smooth muscle cells. Its release is triggered in response to proinflammatory cytokines, such as IL-1β, TNF-α, as well as pathogenic antigen [104]. PTX3 also has known functions in regulating tissue repair through modulation of the extracellular matrix. In previous animal studies, deficiency of PTX3 led to increased fibrin and collagen deposition [105]. Previous studies have demonstrated that patients with PAD and CAD have elevated levels of PTX3 [106], and PTX3 can predict both all-cause mortality and cardiovascular mortality in patients with chronic kidney disease [107]. A study conducted in 116 hemodialysis patients demonstrated that a PTX3 with a cut-off value 3.33 ng/mL had predictive capabilities for PAD (AUC 0.640, *p* < 0.05) and predicted all-cause mortality (HR 1.105, *p* = 0.03) [59].

#### 3.1.13. Anti-Phosphorylcholine IgM

Anti-phosphorylcholine IgM (anti-PC IgM) is an antibody originating from B1 cells, primarily found in the peritoneal and pleural cavities [108]. Anti-PC IgM is primarily involved in the immune response against phosphorylcholine, a component commonly found in bacterial cell walls and oxidized lipids [108]. These antibodies play a critical role in immunological regulation and atheroprotective processes by targeting oxidized lipid components, facilitating the clearance of plaques into the bloodstream [109]. Low anti-PC IgM levels have been linked to an increased risk of cardiovascular diseases, as they help limit inflammation and plaque accumulation [110].

In PAD, anti-PC IgM is hypothesized to have a protective function by reducing inflammation and preventing lipid oxidation in arterial walls, thereby counterbalancing PAD progression [23]. One study analyzed anti-PC IgM as a prognostic biomarker in a cohort of 142 PAD patients undergoing surgery for critical limb ischemia (CLI) [23]. Preoperative anti-PC IgM levels among these patients had a median of 49 units/mL (IQR 32.3–107.7) [23]. A significant association was observed between low anti-PC IgM levels and an increased risk of graft failure (HR 2.11, 95% CI 1.09–4.07, *p* = 0.03) (4). These findings highlight the potential of anti-PC IgM as a valuable biomarker for assessing disease severity and guiding therapeutic strategies in PAD [23].

#### 3.1.14. Galectin-3

Galectin-3 (Gal-3) is a β-galactoside-binding protein from the lectin family. It plays a significant role in various biological activities, including cell adhesion, inflammation, fibrogenesis, and oxidative stress modulation [111]. Gal-3 is primarily expressed in macrophages, contributing to cell proliferation, regulation, and immune defense [112]. It is widely used as a biomarker for cardiovascular and renal diseases, with increased mortality observed in MI and chronic heart failure patients associated with elevated Gal-3 levels [113]. In PAD, Gal-3 is hypothesized to contribute to disease progression through its inflammatory and pro-fibrotic effects on vascular tissue [111]. It is further hypothesized that elevated Gal-3 levels correlate with more advanced PAD due to enhanced macrophage activity, promotion of oxidative stress, and increased cell proliferation, leading to chronic vascular inflammation and arterial remodeling [111]. One study analyzed the association between fibrosis, inflammatory markers such as Gal-3, and their prognostic value in the long-term risk of PAD and CLI [39]. The study, which included 9851 participants without PAD, found a significant association between increased Gal-3 levels and PAD and CLI risk [39]. The hazard ratios were 1.17 (95% CI, 1.05–1.31) for PAD and 1.25 (95% CI, 1.05–1.49) for CLI per unit increase in Gal-3. These results underscore the critical role of Gal-3 in PAD pathophysiology and its potential as a predictive biomarker for disease progression and clinical outcomes [39].

### 3.2. Hemostasis

Complications and adverse clinical outcomes due to PAD are often the result of hypercoagulable states and increased platelet reactivity. Individuals with PAD have an increased risk of MI and CVA due to this hypercoagulable state [114,115]. Increased platelet activation has been associated with PAD diagnoses. Furthermore, platelet volume has been found to increase with PAD. In the National Health and Nutrition Examination Survey (NHANES, 1999–2004), mean platelet volume and PAD had a significant association (*p* = 0.003), even when adjusted for confounders (OR 1.58, 95% CI: 1.14–2.19) [116,117]. A hypercoagulable state has also been shown to increase atherosclerotic plaque formation, worsening the progression and risk of atherosclerotic cardiovascular diseases [118]. This association with PAD and pro-thrombotic vascular events provides the possibility of determining unique proteins involved in hemostasis that may be prognostic markers for PAD. In this review, five proteins were found to investigate the relationship between PAD and coagulation or platelet function.

#### 3.2.1. P-Selectin

P-Selectin is a cell adhesion molecule belonging to the selectin family of proteins that is expressed on activated endothelial cells and platelets, leading to binding and activation of inflammation and hemostasis. P-selectin on endothelial cells also leads to leukocyte recruitment to sites of endothelial injury through binding to the P-selectin glycoprotein ligand-1 (PSGL-1) receptor on leukocytes. P-selectin has been associated with atherosclerotic disease, as it increases leukocyte recruitment at the site of atherosclerotic plaque formation as well as increases the risk of thrombus formation by activating platelets. Studies have demonstrated that patients with PAD have higher levels of P-selectin [119]. In this review, two studies conducted by Gremmel et al. in 2014 investigated if P-selectin could be used as a prognostic marker for PAD. In the first study, 104 patients with PAD, of which 7 had an adverse atherothrombotic event, were compared. P-selectin was elevated in patients with adverse atherothrombotic events but non-significantly (*p* = 0.08) [21]. In the second study, 104 patients were followed post-infra-inguinal angioplasty and stenting for lower extremity artery disease. The researchers found that P-selectin levels above 40.2 mean fluorescence intensity (MFI) by flow cytometry were significantly associated with a 3-fold-increased risk of non-fatal myocardial infarction, ischemic stroke or transient ischemic attack, and recurrent PAD symptoms (95% CI: 1.3–7, *p* = 0.009) [49].

#### 3.2.2. Thrombin

Thrombin is a critical enzyme within the coagulation cascade, leading to the activation of both platelets, as well as the conversion of fibrinogen to fibrin. Thrombin is a procoagulant protein that can activate both platelets and certain coagulation factors, including V, VIII, and XI [120]. Furthermore, it stabilizes hemostatic plugs via the cleavage of fibrinogen and downstream cross-linking of fibrin. It also serves anticoagulant properties via thrombomodulin activation and consequential fibrinolysis. Higher levels of thrombin are often found at sites of vascular injury [121]. Clinically, exogenous thrombin can be used as a hemostatic agent to prevent excessive blood loss during surgery [122]. Thrombin is also highly associated with the inflammatory response and can lead to the expression of cellular adhesion molecules on endothelial cells and the release of proinflammatory cytokines. It is known to exacerbate atherosclerotic plaque formation and vascular lesions [123]. A study of 104 patients with PAD by Gremmel et al. found that thrombin <390 nM consequentially predicted atherothrombotic events with a sensitivity of 85.7% and a specificity of 67% [21].

#### 3.2.3. Glycoprotein IIb/IIIa

Glycoprotein (GP) IIb/IIIa is a glycoprotein which serves as a ligand-binding site for von Willebrand factor (vWf) and fibrinogen on megakaryocytes and platelets. This leads to the aggregation of platelets at sites of tissue injury [124]. Clinically, GPIIb/IIIa is targeted with inhibitors to prevent the aggregation of platelets and fibrinogen binding. It is typically used in the contexts of unstable angina and coronary artery disease (CAD) management [125]. In the vascular literature, a study by Gremmel et al. of 104 patients with PAD undergoing infra-inguinal angioplasty and stenting found that increased GP11b/IIIa levels were associated with a 2.9-fold-increased risk of non-fatal myocardial infarction, ischemic stroke or transient ischemic attack, and recurrent PAD symptoms (95% CI: 1.1–7.5; *p* = 0.04) [49].

#### 3.2.4. Thrombin Receptor Activator Peptide 6 (TRAP-6)

Thrombin-receptor-activating peptide-6 (TRAP-6) is as an agonist for the proteinase-activated receptor 1 (PAR-1) thrombin receptor on platelets and leads to platelet aggregation and consequential hemostatic plug formation mimicking the actions of thrombin [126]. TRAP-6 is often used to evaluate platelet function, known as the TRAP test, and is used to test if platelets will have an expected response to anti-platelet medication [49]. In a study by Gremmel et al. of 104 patients with PAD, high levels of TRAP-6 inducible GP11b/IIIa were associated with a 2.9-fold-increased risk of non-fatal myocardial infarction, ischemic stroke or transient ischemic attack, and recurrent PAD symptoms (95% CI: 1.1–7.5; *p* = 0.04). Further, TRAP-6-inducible high P-selectin levels were significantly associated with a 3-fold-increased risk of these outcomes (95% CI: 1.3–7; *p* = 0.009) [49].

#### 3.2.5. D-Dimer

A D-dimer is a degradation product of fibrin that is a marker of fibrinolysis and ultimately confirms the presence of the hemostatic process [127]. In clinical use, it is often used as a marker to aid the diagnosis of disseminated intravascular coagulation (DIC), pulmonary embolism (PE), and deep vein thrombosis (DVT) [128]. A study by McDermott et al. analyzed 595 participants with PAD and found that D-dimer levels were higher 8 months (*p* = 0.028), 6 months (*p* = 0.005), and 4 months (*p* = 0.017) before any ischemic heart disease (IHD) events (myocardial infarctions, IHD death, or unstable angina) [32]. However, this was not supported by another study by Takamura et al., which evaluated 35 patients undergoing EVT for PAD. It was found that although D-dimer levels did increase from baseline after EVT (*p* < 0.01), there was no association with adverse clinical outcomes, such as limb-related events or death [33].

### 3.3. Kidney Function

Chronic kidney disease (CKD) is known to be associated with the increase risk of atherosclerotic disease and PAD [129]. Both CKD and PAD share risk factors for atherosclerosis, such as hypertension, smoking, diabetes, and hyperlipidemia. However, there is also a studied causal effect of CKD and consequential PAD. This has been hypothesized to be a result of pro-atherosclerotic states in CKD, such as hypoalbuminemia and inflammatory or pro-calcific states [130]. Data from the United States National Health and Nutrition Examination Survey (UH NHANES) have found a 6.5-fold-increased risk of PAD when eGFR is <60 mL/min/1.73 m, even after controlling for confounding baseline characteristics [131,132,133]. When PAD is present in the context of pre-existing CKD, other atherosclerotic endpoints such as limb loss, MA, CVA, and mortality are at an increased risk in prognosis [130,134]. As these are important outcomes to consider in the progression of PAD, determining the relationship of these non-invasively measured biomarkers and adverse PAD outcomes may be beneficial and serve as a suggestion for cost-effective clinical tools for determining prognosis. In this review, two markers of kidney function were found to be investigated for their prognostic capabilities in patients with PAD.

#### 3.3.1. Cystatin

Cystatin is a basic, non-glycosylated protein that is ubiquitous to cells with nuclei. It functions as a regulator of cathepsins in the human vasculature, which are often expressed in atherosclerotic plaques [135]. In the serum, cystatin levels are used as a marker for glomerular filtration, a surrogate of kidney function, and is particularly useful in clinical scenarios where increased muscle mass impairs interpretations of creatinine [136]. The role of serum cystatin and its association with adverse outcomes in PAD is currently unclear in the vascular literature. In a study of 350 patients with PAD by Yang et al., serum cystatin, when increased by ≥5% at 24 h post-contrast medium administration for peripheral arterial angiography, was found to be an independent predictor for 1-year major adverse events (HR: 3.576, 95% CI: 1.354–9.447, *p* = 0.010) [35]. However, in a study of 98 individuals with PAD by Skoglund et al., serum cystatin did not show an association with cardiovascular events [22].

#### 3.3.2. Creatinine

Creatinine is a lactam that is produced when muscle tissue catabolizes creatine [137]. It currently serves as a gold-standard for evaluation of kidney function, as it is primarily filtered out of the body via the glomeruli. Consequentially, serum levels of creatinine are used as a marker of kidney disfunction, as the protein is not cleared from the body [138]. The role of serum creatinine and its association with adverse outcomes in PAD is currently unclear in the vascular literature. In a study of 350 patients with PAD by Yang et al., serum creatinine increase at 24 and 48 h post-contrast medium administration for peripheral arterial angiography was not found to be associated with any 1-year major adverse event (HR: 1.008, 95% CI: 1.000–1.016), *p* = 0.063) [35]. However, in a study by Abbas et al. of 292 female patients undergoing endovascular intervention for PAD, increased pre-intervention creatinine was found to be correlated with endovascular intervention and target vessel revascularization (*p* = 0.015). Also, preintervention creatinine was found to be a significant predictor of mortality (*p* < 0.0001) [61].

### 3.4. Growth Factors and Hormones

Growth factors and hormones play critical roles in pathophysiological processes that are relevant in PAD progression, such as vasculogenesis, inflammation, and tissue remodeling. Dysregulation of growth factors and hormones such as vascular endothelial growth factor (VEGF) or insulin can contribute to the progression of atherosclerotic disease [139,140]. Investigating plasma protein growth factors and hormones may provide insight into new mechanisms of the disease initiation and progression and possible therapeutic targets to increase angiogenesis, reduce progression of atherosclerosis, and reduce adverse outcomes. In this review, four growth factors and hormones were found to be investigated for their prognostic capabilities in patients with PAD.

#### 3.4.1. Insulin-Like Growth Factor-I (IGF-I)

Insulin-like growth factor-I (IGF-I) is a 70-amino-acid peptide hormone primarily produced in the liver under growth hormone (GH) regulation [141]. It plays key roles in growth, tissue repair, and metabolic regulation, along with anti-inflammatory and anti-oxidative properties [141]. IGF-I also influences endothelial repair, vascular smooth muscle cell (VSMC) proliferation, and angiogenesis [142]. Low circulating IGF-I levels have been linked to increased cardiovascular mortality, atherosclerosis progression, and impaired vascular function [142]. In PAD, IGF-I is hypothesized to contribute to vascular health by promoting endothelial function, angiogenesis, and vascular remodeling [143].

In this review, only one study analyzed IGF-I and IGFBP-2 levels in a cohort of 440 PAD patients [46]. The study found no significant association between IGF-I levels and mortality. The hazard ratio for all-cause mortality was 1.06 (95% CI: 0.74–1.54), and for cardiovascular mortality, it was 1.18 (95% CI: 0.7–1.99). These results suggest that IGF-I, despite its biological relevance, may have limited utility as a prognostic biomarker for mortality in PAD [46].

#### 3.4.2. Insulin-Like Growth-Factor-Binding Protein 2 (IGFBP-2)

Insulin-like growth-factor-binding protein-2 (IGFBP-2) is one of six major binding proteins regulating the bioavailability and activity of insulin-like growth factors (IGF-I and IGF-II) [144]. Insulin-like growth factors are crucial for growth, metabolism, and vascular function [145]. Dysregulation of IGFBP-2 has been found to modulate IGF signaling extracellularly and intracellularly, influencing gene expression and cell signaling, leading to various cardiovascular and metabolic diseases [145]. IGFBP-2 has been investigated as a biomarker for cardiovascular disease (CVD), heart failure, and cancer [145,146]. Increased IGFBP-2 levels are associated with disease severity and adverse outcomes, particularly in patients at risk for heart failure and pulmonary arterial hypertension, due to its critical role in vascular remodeling and systemic inflammation [145]. This positions IGFBP-2 as a potential diagnostic and prognostic biomarker.

In PAD, IGFBP-2 is hypothesized to exacerbate disease progression by regulating IGF-mediated processes, such as endothelial repair, smooth muscle cell proliferation, and plaque remodeling [46]. Its relationship with systemic inflammation and cardiovascular dysfunction further supports its potential role as a biomarker in PAD. This review highlights one study that analyzed the prognostic value of IGFBP-2 in cardiovascular mortality. In a cohort of 440 PAD patients, a 100 μg/L increase in baseline IGFBP-2 levels was significantly associated with a higher risk of cardiovascular mortality (adjusted HR 1.12, 95% CI: 1.01–1.24). Despite these results, the receiver-operating curve demonstrated a modest predictive ability, with an area of 0.61 (95% CI: 0.51–0.67, *p* = 0.022) under the curve [46]. This suggests a limited ability for IGFBP-2 to serve as a standalone prognostic marker. Further studies should explore integrating IGFBP-2 with other biomarkers to enhance its clinical utility.

#### 3.4.3. Pregnancy-Associated Plasma Protein-A (PAPP-A)

Pregnancy-associated plasma protein-A (PAPP-A) is a zinc-binding metalloproteinase in the insulin-like growth factor (IGF) system [147]. These proteins cleave IGF-binding proteins, increasing IGF bioavailability and promoting cell proliferation and repair [147]. PAPP-A is linked to vascular inflammation and plaque destabilization, positioning it as a potential biomarker for atherosclerotic diseases [148]. It is particularly associated with inflammatory areas in macrophage and smooth muscle cell regions. Elevated circulating PAPP-A levels are observed in acute coronary syndromes, systemic atherosclerosis, and adverse cardiovascular outcomes. In PAD, PAPP-A is hypothesized to contribute to plaque remodeling and progression by exacerbating vascular inflammation and smooth muscle cell proliferation, linking it to systemic atherosclerosis and vascular dysfunction [148].

This review highlights a study investigating the prognostic role of PAPP-A in 487 symptomatic PAD patients [20]. The study found a significant association between elevated PAPP-A levels and 5-year all-cause mortality (RR 1.31, 95% CI: 1.01–1.73, *p* = 0.024). While this finding underscores PAPP-A’s potential as a long-term prognostic biomarker for PAD, the study’s small sample size and focus on symptomatic PAD patients limit its generalizability [20]. Further research is needed to validate PAPP-A’s prognostic value in broader populations and explore its clinical applications in vascular diseases.

#### 3.4.4. Angiopoietin-Like 2

Angiopoietin-like 2 (ANGPTL2) is a member of the angiopoietin-like protein family, which plays a crucial role in angiogenesis and vascular homeostasis. Within this family, ANGPTL2 is studied for its role in vascular remodeling, while ANGPTL1 is recognized for its role in vessel stabilization [149]. Angiopoietin-like proteins, including ANGPTL2, have also been analyzed as oncological biomarkers and therapeutic targets in tumor angiogenesis, and they are implicated in cardiovascular diseases, such as heart failure and atherosclerosis [150,151]. In PAD, they are hypothesized to influence disease progression through vascular remodeling, inflammation, and endothelial dysfunction. Elevated ANGPTL2 levels are associated with increased vascular permeability and inflammatory signaling, thereby enhancing PAD progression [152].

This review discusses two prospective cohort studies that evaluated the prognostic value of ANGPTL2 in individuals with type 2 diabetes, focusing on the risks of lower-extremity artery disease (LEAD) and peripheral arterial disease (PAD) [17,18]. In the LEAD study of 1412 participants, 112 developed LEAD during the study period [17]. ANGPTL2 levels were significantly associated with the third versus first tertiles (HR 2.04, 95% CI: 1.17–3.57, *p* = 0.01) [17]. The second study examined 1395 participants, of whom 66 required aortic or lower-limb revascularization [18]. ANGPTL2 levels were significantly associated with the need for revascularization (HR 2.16, 95% CI: 1.19–3.92, *p* = 0.01) [18].

### 3.5. Extracellular Matrix Remodeling

The extracellular matrix (ECM) plays an important role in vascular structure and remodeling. It also plays a pivotal role in the progression of atherosclerotic disease. These proteins influence the structure of the vasculature by regulating processes such as collagen breakdown, elastin degradation, and tissue repair [153]. Imbalances between ECM proteins play a pathological role in several diseases, including coronary artery disease and abdominal aortic aneurysms, and investigating their potential as markers of PAD prognosis [154,155]. In our review, two proteins with functions in extracellular matrix remodeling were found to be investigated for PAD prognosis.

#### 3.5.1. Matrix Metalloproteinase-10

Matrix metalloproteinase-10 (MMP-10) is a member of the matrix metalloproteinase family, responsible for degrading extracellular matrix (ECM) components such as proteoglycans, collagen, and elastin [156]. MMP-10 is expressed in inflammatory cells and vascular smooth muscle cells, playing a crucial role in ECM remodeling, inflammation, and angiogenesis [156,157]. It is also involved in vascular remodeling and atherosclerotic plaque development [157]. Recent studies have associated MMP-10 with cardiovascular diseases and inflammatory conditions that contribute to ECM degradation and vascular remodeling [157,158]. Elevated MMP-10 levels have been linked to diseases such as atherosclerosis, chronic kidney disease, and diabetes [159].

In PAD, MMP-10 is hypothesized to contribute to disease progression by facilitating ECM breakdown and promoting inflammatory cell responses. It is also hypothesized to play a crucial role in atherosclerotic plaques and vascular remodeling [158]. This review highlights one study that analyzed MMP-10 levels in 187 PAD patients and 200 sex-matched controls [56]. The study reported significantly elevated circulating MMP-10 levels in PAD patients, with higher levels observed in those with critical limb ischemia (CLI) (1086 ± 478 pg/mL vs. 822 ± 436 pg/mL; *p* < 0.001). Additionally, the univariate analysis found a significant association between elevated MMP-10 levels and both all-cause and cardiovascular mortality (*p* < 0.03) [56]. These findings suggest that MMP-10 could serve as a biomarker for PAD, particularly in advanced cases, such as CLI, but further research is needed to validate its clinical utility.

#### 3.5.2. Tissue Inhibitor of Metalloproteinase (TIMP)

Tissue inhibitor of metalloproteinases-1 (TIMP-1) is a glycoprotein that regulates matrix metalloproteinases (MMPs), enzymes that play a critical role in ECM degradation and the maintenance of ECM integrity [160]. This regulation impacts tissue remodeling, inflammation, and angiogenesis processes [160]. TIMP-1 has been associated with various cardiovascular and inflammatory diseases [161]. Recent studies have found that TIMP-1 levels are elevated in vascular injuries, including arterial stiffness, atherosclerosis, and heart failure [162]. TIMP-1’s role in ECM maintenance makes it a key mediator in vascular homeostasis [162].

In PAD, TIMP-1 is hypothesized to regulate ECM remodeling and inflammatory responses in ischemic and atherosclerotic tissues [161]. Dysregulation of the TIMP/MMP system is also thought to promote plaque instability, impaired angiogenesis, and chronic vascular inflammation, all hallmarks of PAD [161]. This review highlights one study that investigated the association between MMPs and disease severity and mortality in a cohort of 187 PAD patients and 200 sex-matched controls [56]. While the study prioritized MMP-10, it also noted an association between elevated TIMP-1 levels and PAD severity, particularly in patients with critical limb ischemia (CLI) [56]. Although TIMP-1 shows promise as a biomarker in PAD, its utility is limited by a lack of specificity and the complex interplay between TIMP-1 and MMPs [56].

### 3.6. Metabolism

Primarily, PAD is an atherosclerotic disease that reduces blood flow to the peripheral arteries. However, it has more recently been identified to have a heterogeneous causes with oxidative stress and systemic dysfunction due to endothelial dysfunction and metabolism dysfunctions within skeletal muscle being significant influencers of disease progression [163,164]. Furthermore, markers of metabolic dysfunction, such as apoliproteins, have shown a strong association with PAD [165]. In this review, four markers of metabolism were found to have been investigated as potential markers of PAD prognosis.

#### 3.6.1. Low-Density Lipoprotein

Low-density lipoprotein (LDL) cholesterol is a circulating lipid that is produced when free fatty acids are stored as triacylglycerols in adipose tissue. The process of producing triacylglycerol from free fatty acids requires the release of LDL [166]. At increased levels, typically from exogenous fatty acid sources, such as high saturated fatty acid diets, hypercholesterolemia can result. This process increases the risk for atherosclerotic disease via the deposition of oxidized LDL within the endothelium of the vasculature, which leads to an inflammatory response [167]. Clinically, serum LDL is measured to establish risk of future cardiovascular disease [168]. In a study by Aday et al., 27,888 women free of cardiovascular disease at baseline were followed for 15.1 years. It was found that high levels of total and small LDL particles were associated with increased PAD risk. (2.03; 95% CI, 1.14–3.59) (2.17; 95% CI, 1.10–4.27) [53].

#### 3.6.2. High-Density Lipoprotein

High-density lipoprotein (HDL) cholesterol is a circulating lipoprotein that is made up of triglycerides, cholesterol, and apo-lipoproteins. It serves to transport cholesterol throughout the body, typically from peripheral tissues and cells to the liver for degradation and removal [169]. Clinically, increased fasting serum HDL levels are considered to be protective of the vasculature and tested to better inform physicians about risks for cardiovascular disease [170]. The SVS guidelines support this clinical use [2]. In a study of 254 patients with PAD or CLI (critical limb ischemia) by Martinez-Aguilar et al., it was found that patients with normal levels of HDL had reduced incidence of mortality (HR 0.34, 95% CI: 0.21–0.57) [19]. In another study by Aday et al., 27,888 women free of cardiovascular disease at baseline were followed for 15.1 years, and HDL particles were found to have an inverse relationship with PAD risk (0.29; 95% CI, 0.16 to 0.52; P trend < 0.0001) [168].

#### 3.6.3. Malondialdehyde-Modified Low-Density Lipoprotein (MDA/LDL)

Malondialdehyde-modified low-density lipoprotein (MDA/LDL) is an oxidized derivative of LDL that is created during the process of plaque formation. Circulating MDA/LDL is elevated in states of oxidative stress and consequential atherosclerotic cardiovascular disease [171]. In a study by Takamura et al. of 35 patients undergoing endovascular thrombectomy (EVT) for PAD, MDA/LDL ratios (comparing post- to pre-EVT values) divided participants into high (≥0.495) and low (<0.495) cohorts. The low-ratio cohort, indicating sustained high MDA/LDL levels post-EVT, had more limb-related events or death (*p* < 0.001), and associations with clinical endpoints, such as major adverse cardiovascular events (MACEs), major adverse limb events (MALEs), and major adverse cardiovascular and limb events (MACLEs) (HR 0.4210, *p* = 0.0154) [33].

#### 3.6.4. Homocysteine

Homocysteine is an amino acid that is formed as a derivative of methionine, an essential amino acid, when it is metabolized to cysteine. Circulating levels of homocysteine are typically elevated in pathologies such as CVA, cardiovascular disease, aortic aneurysms, and end-stage renal disease [172]. Despite the association of homocysteine to arteriosclerotic diseases, it is typically used in a clinical setting to evaluate vitamin deficiencies due to an inverse relationship of homocysteine levels with folate, B6, and B12 [173]. In the vascular literature, a study of 556 PAD patients by Amrock et al. demonstrated that elevated homocysteine was found to be strongly correlated with all-cause mortality (HR 1.31, 95% CI: 1.11–1.54, *p* < 0.001) [57].

### 3.7. Cardiac Markers

Cardiac biomarkers provide insight into various physiological and pathological processes, including oxidative stress, myocyte injury, and inflammation [174]. These biomarkers reflect the health of the cardiovascular system and can serve as early indicators of disease, monitor disease progression, and could help predict outcomes in vascular diseases, such as PAD [174,175]. Risk factors for PAD are similar to other cardiovascular diseases, including hypertension, smoking, and diabetes mellitus [133]. While traditional risk factors remain critical, cardiac biomarkers may enhance our understanding of PAD’s pathophysiology and help improve its management.

#### 3.7.1. Brain Natriuretic Peptide

B-type natriuretic peptide (BNP) is a peptide hormone released into circulation by ventricular cardiomyocytes in response to wall stress caused by volume or pressure overload [174,176,177]. It helps regulate blood pressure and volume by promoting vessel dilation, increasing sodium and water excretion by the kidneys, and suppressing the renin–angiotensin–aldosterone system [178]. The clinical utility of BNP as a biomarker for diagnosing and assessing cardiac function have been well documented [179,180,181]. In two studies evaluating the prognostic value of BNP in patients with PAD, those with elevated levels of BNP had significantly greater risks of cardiovascular events [25,182]. One study demonstrated that patients with elevated pre-operative BNP levels exhibited a 10.6-fold increase in risk (95% CI: 2.6–42.9, *p* = 0.001) of major cardiovascular events [182]. These findings indicate that BNP holds promise as a marker for identifying PAD patients at increased risk of future cardiovascular events. However, further research is needed to validate these results and explore their clinical implications.

#### 3.7.2. N-Terminal Prohormone of Brain Natriuretic Peptide

BNP is initially synthesized as the precursor hormone preproBNP in myocytes, which is cleaved in circulation to produce its active form, BNP, and its inactive form, N-terminal pro-B-type natriuretic peptide (NT-proBNP) [178]. Recent studies have also demonstrated the prognostic value of BNP and NT-proBNP in evaluating systemic vascular dysfunction, ischemia, and cardiovascular stress in PAD, serving as potential markers for disease severity, progression, and cardiovascular risk [22,24,36]. Research on NT-proBNP from three studies has demonstrated its significant association with cardiovascular outcomes in PAD [22,24,36]. One study identified that NT-proBNP was a key predictor of 3-year major adverse limb events (MALEs), with its inclusion in a machine learning model improving predictive accuracy for adverse outcomes in PAD patients [36]. Another study, comparing NT-proBNP concentrations measured by different assays, showed that NT-proBNP values reliably predicted future cardiovascular events, with similar discriminatory accuracy between conventional and proximity extension assays (tdAUC = 0.65–0.66) [24]. This shows that NT-proBNP can serve as a robust prognostic biomarker for assessing long-term cardiovascular risk in PAD patients. Together, these findings highlight the potential of integrating NT-proBNP measurements into routine risk assessment for PAD patients, which may facilitate earlier interventions and improve patient outcomes.

#### 3.7.3. Cardiac Troponin T (cTnT)

Cardiac Troponin T (cTnT) is a myofibrillar protein within the troponin complex of cardiomyocytes that modulates contraction by regulating the interaction between actin and myosin in response to changes in intracellular calcium concentrations [183]. It plays an important role in regulating the initiation and inhibition of contraction to maintain the contractile function of the heart, ensuring appropriate cardiac output [183]. Damage to cardiomyocytes leads to an increase in cTnT in the blood, making cTnT a biomarker routinely used as a measurement of cardiomyocyte injury and stress in PAD [174,184]. The Cardiovascular Disease in Intermittent Claudication (CAVASIC) study investigated whether baseline high-sensitivity (hs)-cTnT was associated with increased risk of cardiovascular events and all-cause mortality in male patients with PAD [42]. The study showed that detectable hs-cTnT was associated with an 84% higher likelihood of symptomatic PAD at baseline, even when adjusted for NT-proBNP. Moreover, elevated hs-cTnT levels (≥14 ng/L) were strongly predictive of incident of CVD and all-cause mortality in PAD patients. Specifically, patients with hs-cTnT ≥ 14 ng/L had a significantly increased risk of incident CVD (Hazard ratio (HR) = 3.15, *p* = 0.01) and an elevated risk of all-cause mortality (HR = 5.06, *p* < 0.001) [42]. These findings suggest that hs-cTnT could be an indicator of ongoing myocardial stress in patients with symptomatic PAD. However, since this study was conducted in a male-only cohort, further research in diverse populations is necessary to validate these results.

#### 3.7.4. Ischemia-Modified Albumin (IMA)

Ischemia-modified albumin (IMA) forms during oxidative stress, which damages the N-terminal region of the albumin protein and impairs its ability to bind transition metals [185]. PAD is characterized by reduced blood flow due to narrowing of the arteries, which can result in tissue ischemia [186]. Elevated levels of IMA in individuals with PAD could be reflective of underlying ischemia and oxidative stress, making it a potential biomarker for PAD progression. A recent study in the SURDIAGENE cohort assessed IMA and other inflammatory and redox biomarkers in individuals with type 2 diabetes to predict lower-extremity artery disease (LEAD) [17]. High baseline IMA levels were independently associated with a 5.6-fold-increased risk of major LEAD, defined by peripheral revascularization or lower-limb amputation. In another study, plasma concentrations of biomarkers such as TNF receptor 1 (TNFR1), fluorescent advanced glycation end products (F-AGEs), IMA, and total reductive capacity of plasma (TRCP) were evaluated for their predictive value in PAD among individuals with type 2 diabetes [18]. HR for the highest tertiles versus the lowest were significant for TNFR1 (HR: 1.59, *p* < 0.0001), F-AGE (HR: 2.00, *p* < 0.0001), and TRCP (HR: 1.25, *p* = 0.002) but not for IMA. Further research is needed to fully elucidate IMA’s prognostic value for PAD.

#### 3.7.5. Hemoglobin A1c and Glycosylated Hemoglobin

Hemoglobin is an essential protein in erythrocytes that transports oxygen throughout the body. Glycated or glycosylated hemoglobin (HbA1c) forms when glucose in the blood binds to hemoglobin through a process called glycation [187]. HbA1c serves as an indicator of overall glycemic control, used to determine the average blood sugar levels over three months, with levels above 6.5% indicating a diagnosis of diabetes mellitus (DM) [187,188]. PAD, often associated with DM, involves arterial narrowing and impaired blood flow, which exacerbates oxidative stress and vascular inflammation. Elevated HbA1c levels in individuals with PAD may indicate poor glycemic control, contributing to endothelial dysfunction and atherosclerosis, making it a potential biomarker for PAD progression and associated complications. Recent evidence has demonstrated a significant association between elevated HbA1c levels and an increased risk in developing PAD and associated complications [16,40,41,47,48,51,58,60,64]. For instance, the Atherosclerosis Risk in Communities study demonstrated that HbA1c ≥ 7% were found to significantly elevate the risk of developing PAD (HR 6.00, 95% CI: 3.73–9.66) and critical limb ischemia (CLI) (HR 10.39, 95% CI: 4.79–22.53) [64]. Similarly, research on Japanese patients with end-stage renal disease demonstrated that HbA1c ≥ 51 mmol/mol were associated with a greater incidence of PAD (HR 1.63, 95% CI 1.17–2.28) and limb amputation (HR 2.99, 95% CI 1.17–7.70) underscoring the role of HbA1c as a marker for assessing the risk of PAD development [47].

Some studies have also examined the role of preprocedural HbA1c levels in predicting PAD treatment outcomes. Poor glycemic control has been associated with higher rates of MALEs and amputation across various interventions [51,60]. For example, diabetic men undergoing endovascular therapy with HbA1c ≥ 7% had more MALEs than those with HbA1c < 7.0% [51]. Similarly, a large cohort study of 30,813 patients undergoing infra-inguinal bypass surgery found that poor glycemic control was significantly associated with an increased risk of major limb amputation and postoperative complications [60].

Additionally, studies have shown that higher HbA1c levels are linked to worse outcomes following revascularization procedures [16,41,48]. For example, patients with HbA1c > 8% had a 105% increased risk of amputation compared to those with HbA1c ≤ 6% and had significantly higher odds of adverse limb events (OR 1.46, 95% CI: 1.07–2.00, *p* = 0.04) [15,41]. Preoperative HbA1c levels > 6.5% have also been associated with higher rates of 30-day hospital readmission (OR 1.06, 95% CI: 1.00–1.12, *p* = 0.04) [16]. Lastly, those with HbA1c > 8% have experienced significantly higher risk of complications, such as restenosis (35.48% vs. 9.09%, *p* = 0.03), delayed ulcer healing (16.13% vs. 45.0%, *p* = 0.02), and reduced quality of life metrics following a procedure further emphasizing the importance of managing glycemic control [48].

In terms of mortality, research indicates a possible association between elevated HbA1c and higher all-cause mortality in PAD patients [40,58]. One study found that older adults with HbA1c > 7% had a significantly higher risk of mortality (HR 1.3, 95% CI: 1.04–1.63), while another demonstrated a significant association between HbA1c levels and all-cause mortality (HR: 1.75, 95% CI: 1.24–2.46, *p* = 0.01) [40,58]. This association remained significant after adjusting for age, sex, and medication use (HR: 1.54, 95% CI: 1.03–2.32, *p* = 0.04), though it became nonsignificant after full adjustment (HR: 1.39, 95% CI: 0.92–2.30, *p* = 0.13) [40].

Interestingly, some studies have shown that elevated HbA1c levels may have a protective effect in certain subgroups. For instance, in patients with critical limb ischemia (CLI) undergoing lower-extremity bypass, elevated HbA1c (>6.5%) was associated with a reduced risk of death within one year (HR 0.75, 95% CI: 0.61–0.93, *p* = 0.01) [63]. Similarly, patients scheduled for lower-extremity amputation with perioperative HbA1c values between 8.5 and 9.4% (HR 0.57, 95% CI: 0.35–0.93) and ≥9.5% (HR 0.46, 95% CI: 0.31–0.69) had a lower risk of mortality compared to those with HbA1c < 8.5%, although these results were not significant after adjusting for age and sex [37]. Further, some studies have demonstrated that there is no significant difference between HbA1c levels and risk of developing complications. There was no significant difference in HbA1c levels between symptomatic PAD patients who experienced major adverse cardiovascular events (MACEs) and those who did not [14]. Similarly, in patients with type 2 diabetes, HbA1c levels were not significantly different amongst patients who developed major LEAD, aortic revascularization, or limb revascularization and those who did not [14,18].

Together, these findings highlight the complex relationship between HbA1c and PAD outcomes. While elevated HbA1c generally indicates worsening, including higher risks of amputation and complications, further research is needed to determine its prognostic role in specific subgroups

### 3.8. Transport Proteins

Specific transport proteins are essential for the movement of critical nutrients, molecules, and ions, supporting nearly all physiological functions [17]. In the vascular system, these proteins maintain homeostasis by distributing vital substances, including hormones, fatty acids, metals, and proteins, to various parts of the body. When these transport mechanisms become dysregulated, they can lead to an excess or shortage of necessary molecules, potentially contributing to the onset of vascular conditions [17]. Certain transport proteins that regulate ions and other molecules have been linked to the development and progression of PAD.

#### 3.8.1. Albumin

Albumin, the most abundant protein in plasma, plays a crucial role in various physiological processes [189]. Its primary functions include maintaining oncotic pressure, transporting molecules such as hormones and fatty acids, buffering blood pH, and facilitating the binding and detoxification of waste products. Synthesized and excreted by the liver at a rate of 10–15 g per day, albumin serves as a key indicator of liver function [190].

Low levels of serum albumin have been associated with increased systemic inflammation and heightened risk of adverse cardiovascular events [191]. Moreover, albuminuria has shown independent associations with left ventricular mass and greater carotid intima–media thickness in both diabetic and non-diabetic individuals [192,193]. These findings support a potential pathophysiologic link between albuminuria and PAD [194].

Recent studies have identified serum albumin levels <2.5 g/dL as a significant marker for increased risk of mortality and unplanned reoperations [12,43]. Specifically, in patients with PAD, low serum albumin levels predicted 1-year major adverse events [35]. Further, one study identified glycated albumin, a nontraditional glycemic marker, as an independent factor linked to PAD development. Its levels reflect glucose metabolism and are associated with PAD progression, offering valuable insight, particularly in the progression of CLI [64]. Serum albumin levels were also significant independent predictors of major adverse cardiovascular events (HR 0.55, 95% CI: 0.38–0.79, *p* = 0.0014) and major adverse limb events (HR 0.59, 95% CI: 0.36–0.95, *p* = 0.030) [13].

A study evaluating hospitalization rates demonstrated that severe hypoalbuminemia was associated with higher rates of 30-day readmission (*p* = 0.005), 90-day ER visits (*p* = 0.006), and 90-day readmission (*p* = 0.001) rates compared to moderate hypoalbuminemia and normal albumin levels [44]. Severe hypoalbuminemia was independently associated with a greater likelihood of 90-day ER visits (OR = 2.8, 95% CI, 1.23–6.36, *p* = 0.014) and 90-day readmission (OR = 2.63, 95% CI, 1.21–5.71, *p* = 0.015). Serum album < 2.8 g/dL demonstrated significantly higher prolonged length of hospital stay as well (adjusted means ratio 1.2, 95% 1.1–1. 2, *p* < 0.001) when compared to individuals with serum album ≥ 3.5 g/dL [45].

#### 3.8.2. Hemoglobin

Hemoglobin is an essential protein in erythrocytes that transports oxygen throughout the body. Low hemoglobin levels have been associated with worse outcomes in patients with PAD, as reduced oxygen-carrying capacity can exacerbate limb ischemia and contribute to disease progression [54]. One study identified low hemoglobin as an independent risk factor for MACEs (OR for each 1 g/dL drop below the mean = 1.4 [1.13–1.7]; *p* = 0.002) and death (OR for each 1 g/dL drop below the mean = 1.5; 95% CI [1.14–1.86]; *p* = 0.002) [54]. Additional research of hemoglobin levels in PAD patients is required to evaluate the prognostic value of hemoglobin in PAD.

#### 3.8.3. Fatty-Acid-Binding Protein-3 (FABP3)

Fatty-acid-binding protein 3 (FABP-3) binds long-chain fatty acids and facilitates their transport within cells [195]. These fatty acids are critical for various cellular processes, including providing energy to cardiomyocytes. FABP-3 is currently used as a biomarker for identifying acute myocardial infarctions [196]. When cardiomyocytes are damaged, they release FABP-3 into circulation [195,196]. Recent evidence has also linked FABP-3 to PAD progression. Elevated FABP3 levels may indicate muscle damage or ischemia resulting from impaired circulation [195]. One recent study evaluating prognostic markers for PAD, developed prediction models for 3-year PAD-related MALEs [36]. FABP-3 was observed to have the greatest predictive importance among the 10 biomarkers used in the predictive equation for forecasting 3-year MALEs in patients with PAD. Its elevated levels were strongly associated with increased risk, highlighting its potential as a biomarker for identifying high-risk PAD patients and guiding clinical interventions aimed at reducing complications such as limb loss or critical ischemia.

#### 3.8.4. Fatty-Acid-Binding Protein-4 (FABP4)

Fatty-acid-binding protein 4 (FABP-4) is primarily expressed in adipocytes and macrophages where it facilitates transport and is found to be highly induced during adipocyte differentiation [197,198]. FABP4 has been linked to the biological development of atherosclerosis by contributing to inflammation, insulin resistance, and other cardiovascular risk and has shown potential as a biomarker for diagnosing PAD in diabetic patients [198,199]. In a recent study evaluating FABP-4 prognostic value in PAD, it was found that each one-unit increase in FABP-4 was significantly associated with MALEs (HR 1.18, 95% CI 1.03–1.27; *p* = 0.022) and worsening PAD (adjusted HR 1.17, 95% CI 1.12–1.28; *p* < 0.001) [38]. Similarly, another study observed that FABP-4 was a significant predictor of 3-year MALE in PAD patients [36]. These findings emphasize the potential clinical utility of FABP-4 in risk stratification and prognosis for PAD patients, highlighting its relevance in both short- and long-term disease management.

#### 3.8.5. Adipocyte Fatty-Acid-Binding Protein (A-FABP)

Adipocyte fatty-acid-binding protein (A-FABP) is lipid-binding protein that is predominantly expressed in adipose tissue involved in lipid metabolism and inflammation [200]. Elevated A-FABP levels are associated with the development of T2D and atherosclerosis, contributing to oxidative stress and arterial stiffness, key factors in PAD progression [200]. A study in T2D patients found higher serum A-FABP levels in those with PAD, suggesting its potential as a biomarker [11]. Gender differences were observed, with females showing a higher stenosis rate in the highest A-FABP tertile. A-FABP was an independent risk factor for PAD in female T2DM patients but not males. Further research is needed to confirm its clinical utility in PAD progression.

#### 3.8.6. Retinol-Binding Protein 4 (RBP-4)

Retinol-binding protein (RBP-4) is an adipokine with the greatest expression in the liver, where it aids in transport of retinol in circulation [201]. In individuals with diabetes, RBP-4 has demonstrated an association with the development of atherosclerotic risk factors, including inflammation [202]. The association between RBP-4 levels and PAD has been not been fully evaluated [203]. One study evaluating the prognostic role of RBP-4 in 168 symptomatic PAD patients demonstrated that high RBP4 levels were independent predictors of PAD even after adjustment for age [14]. Further after adjustment of cardiovascular risk factors RBP-4 levels remained a significant independent predictor of MACE in PAD patients. RBP-4 could serve as a prognostic indicator of PAD; however, further research is required to demonstrate its utility in understanding disease progression.

### 3.9. Non-Traditional Glycemic Markers: Fructosamine, Glycated Albumin, and 1,5-Anhydroglucitol

As previously discussed, HbA1c serves as a common indicator of overall glycemic control. However, other non-traditional glycemic markers, such as fructosamine, glycated albumin, and 1,5-anhydroglucitol, may also demonstrate utility as prognostic indicators of PAD.

Fructosamine is a stable ketoamine which is formed as a byproduct from the reaction between glucose and an amino group of a albumin, lipoproteins, or other globulins [204]. Similarly, glycated albumin is a specific glycation product formed when glucose irreversibly binds to albumin, representing a subset of the total fructosamine pool [204,205]. Unlike HbA1c, which reflects long-term glycemic control over 2–3 months, glycated albumin provides an intermediate-term measure, reflecting average glucose levels over approximately 2–3 weeks due to the shorter half-life of albumin [205].

Fructosamine and glycated albumin are valuable in clinical contexts where HbA1c measurements may be unreliable [205]. Additionally, these markers have been shown to correlate with the severity of diabetic complications, including PAD, making them potential prognostic indicators for this condition [64]. For instance, one study demonstrated that higher fructosamine and glycated albumin demonstrated increased HR for PAD and CLI, particularly with patients diagnosed with diabetes and those with a higher glycemic index, and remained significant, even after adjusting for cardiovascular risk factors [64]. The greater HR for CLI compared to overall PAD suggests that these markers could be helpful in identifying patients with a greater risk of severe forms of PAD.

Interestingly, a J-shaped association between these markers and PAD and CLI risk were observed, highlighting that intermediate levels may pose less risk than very low or high levels [64]. While further adjustment for fasting glucose and HbA1c attenuated some associations, glycated albumin consistently showed a significant relationship with PAD in certain models.

1,5-Anhydroglucitol (1,5-AG), on the other hand, is a monosaccharide and inversely reflects short-term glycemic fluctuations [206]. Decreased levels of 1,5-AG occur in the presence of sustained hyperglycemia, as renal glucose reabsorption competitively inhibits 1,5-AG reabsorption [206]. This marker is an indicator of short-term glycemic control, which is a known risk factor for vascular complications, including PAD [64]. Compared to fructosamine and glycated albumin, 1,5-AG may provide complementary information but showed attenuated associations with PAD and CLI when adjusted for HbA1c, suggesting that its utility may be context-specific [64].

## 4. Discussion

This comprehensive review was conducted to determine current research on plasma protein biomarkers that were investigated for their prognostic capabilities in patients with PAD. Studies that investigated plasma protein biomarkers and their association with events such as incident peripheral arterial disease, increase in severity, need for surgical intervention, adverse cardiovascular events, such as MI and CVA, and adverse limb events, including minor and major amputation, were included. In the final analysis, 55 studies were found with the inclusion criteria, in which 44 unique proteins were investigated. These proteins had a wide array of functions and were involved in many physiological pathways, including inflammation and immunity, hemostasis, extracellular matrix remodeling, and metabolism, demonstrating the heterogeneity of the disease process. Atherosclerosis is known to be a disease that affects multiple physiological processes; however, inflammation plays a key role in its initiation and progression. Evidently, proteins in this pathway were the most investigated, as found in this review. Specifically, 15 of the 44 proteins had functions within immunity and inflammation.

Currently, targeting inflammation for the prevention of the progression of PAD is being studied, with some studies showing disappointing results. Previous studies have attempted to therapeutically target proteins such as IL-6 and P-Selectin in other cardiovascular diseases with little result [207,208]. Recently, low-dose colchicine therapy has demonstrated potential as a therapeutic drug for the prevention of adverse limb-related events. In a study of 60,000 patients with PAD with an average follow up of 4.5 years, there was a reduction in MALEs by 25%. MACEs were also lower in the group that received colchicine treatment [209]. These studies demonstrate the potential for novel treatment tools for PAD. Some of the markers discovered in this review could also be targets for medical therapy for the prevention of adverse events in patients with PAD. Further research is needed in order to establish a clinically relevant and accepted marker for the prognostication of PAD, as well as potential novel targets for treatment.

Treatment and surgical strategies can vary among patients, and strong clinical biomarkers are needed for prognostication of PAD in order to provide tailored treatment plans. These biomarkers could play a role in deciding if patients should receive endovascular treatment versus open arterial bypass. The Society for Vascular Surgery guidelines state that endovascular treatment should be the first-line treatment provided to patients; however, the long-term efficacy of endovascular treatment is less strong [10]. They state that the decision of which surgery to provide to patients should be individualized to each patient considering not only clinical and anatomical factors but risk of complications such as periprocedural risk, risk of cardiovascular complications, and mortality. For example, patients with aortoiliac occlusive disease can be provided with direct aortic reconstruction (aortofemoral bypass) or aortoiliac endarterectomy, but these carry a higher risk of morbidity and mortality. These, however, are known to be more stable long-term. Those that are at a higher risk may be offered other options, such as an iliac femoral bypass. These provide less immediate risk; however, they are often less durable over time (6). This demonstrates the need for new clinical markers that can assist with determining peri-operative or long-term risk in this patient population. Markers that are disease-specific may be most beneficial to capture the underlying physiological mechanisms of disease manifestation.

Among the many protein biomarkers that were investigated in this review, we believe that LCN2, GDF-15, and Galectin-3 may provide the most promising candidate for proteins to be integrated into clinical use. This is due to their strong prognostic values and specificity for PAD-related outcomes. LCN2 is an adipocyte involved with vascular inflammation as well as atherosclerotic disease and has been associated with MALE and MACE (Table 1) and could potentially be used for risk stratification for earlier intervention. GDF-15, a stress-responsive cytokine linked to oxidative stress and vascular dysfunction, has been consistently associated with increased risk of PAD progression and MACE, contributing to its potential to be included as a relevant prognostic marker for PAD [210]. Laster Galecting-3 is a regulator of inflammation and fibrosis and has been shown to correlate with PAD severity and critical limb ischemia (CLI), indicating its potential as a marker for disease progression, risk of CLI, and a potential target for therapeutic monitoring [211]. Unlike certain markers, such as CRP, which lack disease specificity, these three biomarkers have been linked to disease pathophysiology and specificity for PAD, which may increase their clinical relevance. Current studies investigated have demonstrated a variety of methodologies, including prospective and retrospective cohort studies, case–control studies, and randomized clinical trials. The heterogeneity of the methodology make more direct comparisons difficult. Further studies could integrate proteomics to allow for large-scale profiling of proteins, which may indicate novel proteins to investigate. Also, further research is needed in order to validate their prognostic capabilities in larger studies and integrating them into currently available risk assessment models and determine their accuracy in predicting adverse outcomes in patients with PAD.

With the recent introduction of machine learning into clinical care, accuracy of prognostication has increased. Machine learning can integrate multiple biomarkers and use those features to more accurately predict adverse outcomes compared to each feature alone. This possibility is promising for the use of biomarkers for prognostication, as you can now integrate multiple biomarkers with disease characteristics, clinical characteristics, and past medical history for more accurate prognostication. Recent studies have demonstrated this capability. A study conducted by Li et al. in 2024 used 112 predictive features, which included clinical characteristics, disease characteristics, and biomarkers such as creatinine. They were able to predict 1-year MALE with AUROC of 0.94 (95% CI, 0.93–0.95) and an accuracy of 0.86 (95% CI, 0.85–0.87) [36]. Combining biomarkers, such as LCN2, GDF-15, and Galectin-3, with other disease features has shown promise for better prognostic tools for physicians. There is currently a lack of machine learning models that are clinically relevant and that combine PAD specific biomarkers with other clinical characteristics to predict outcomes in these patients. Further research is required to find suitable biomarkers and models that can fit this need in clinical care.

PAD can often be underdiagnosed and mismanaged, especially within more rural areas where diagnostic and prognostic tools, such as trained ultrasound techs, are not readily accessible [212]. This can lead to delayed treatment and increased risk of adverse events, including limb amputations [212]. Biomarkers that are clinically accepted can be used as markers for monitoring patients who are at risk of PAD to ensure timely medical and surgical management. Blood testing can be more readily accessible within family practice and rural clinics to flag patients who may require further testing and referral to vascular specialists.

There are a few limitations of this study. We only included studies that looked at plasma protein biomarkers. Many other biomarker types have been shown to be strong contenders of prognostication, including urinary markers, microRNA (mRNA) markers, and tissue markers. Three major databases were used: MEDLINE, Embase, and Cochrane CENTRAL libraries; however, some papers may not have been found within these databases. In order to prevent the loss of relevant research articles, each article was reviewed by at least two reviewers. Lastly, this systematic review was not registered before initiation with PROSPERO.

## 5. Conclusions

In this systematic review of the literature, current research on protein plasma biomarkers for the prognosis of PAD were outlined. There is still no marker that is specific for PAD and that is clinically accepted as a biomarker for the prognosis of PAD. There is a need for a marker, or a combination of markers, that can be used to risk-stratify patients for better patient management and personalized treatment plans. Further studies may investigate new markers, or a combination of markers, integrated with clinical characteristics to improve prognostic capabilities.

## Figures and Tables

**Table 1:** Primary research articles published between January 2010 and May 2024 focusing on prognostic protein blood biomarkers for peripheral arterial disease (PAD).

Authors	Proteins	Findings
Ding M, Shi J-Y, Xing Y-Z, Sun B, Fang Q-H, Zhang J-Y et al., 2017 [11]	Adipocyte fatty acid-binding protein (A-FABP)	Women in the third tertile of A-FABP values had significantly higher rates of stent stenosis compared to the first tertile. This trend was not found in men.
Morisaki K, Matsumoto T, Matsubara Y, Inoue K, Aoyagi Y, Matsuda D et al., 2016 [12]	Albumin	Serum albumin < 2.5 g/dL was significantly associated with risk of two-year mortality (HR 3.45, 95% CI: 1.01–11.7, *p* = 0.04).
Ishii H, Aoyama T, Takahashi H, Kamoi D, Tanaka M, Yoshikawa D et al., 2013 [13]	Albumin and C-reactive protein (CRP)	Serum albumin (HR 0.55, 95% CI: 0.38–0.79, *p* = 0.0014) and CRP (HR 1.01, 95% CI: 1.00–1.02, *p* = 0.0022) were significant independent predictors of major adverse cardiovascular events. Similarly for major adverse limb events. Serum albumin (HR 0.59, 95% CI: 0.36–0.95, *p* = 0.030) and CRP (HR 1.01, 95% CI: 1.00–1.02, *p* = 0.040) were significant independent predictors.
Kadoglou NPE, Korakas E, Karkos C, Maratou E, Kanonidis I, Plotas P et al., 2021 [14]	Glycated hemoglobin (HbA1c), Retinol-Binding Protein-4 (RBP-4), adiponectin, high-sensitivity C-Reactive Protein (hsCRP)	RBP4 (β = 0.498, *p* < 0.001) and adiponectin (β = −0.288, *p* < 0.001) levels were independent predictors of PAD (R2 = 0.422, *p* < 0.001) after adjusting for age. After adjusting for cardiovascular risk factors, RBP4 levels remained a significant independent predictor of MACE (β = 0.455, *p* < 0.001).
Singh N, Zeng C, Lewinger JP, Wolfson AM, Shavelle D, Weaver F et al., 2019 [15]	HbA1c	Patients with HbA1c > 8% had a higher odds of an adverse limb events compared to those with HbA1c, between 6 and 7% (OR 1.46, 95% CI: 1.07–2.00, *p* = 0.04).
Buelter J, Smith JB, Carel ZA, Kinsey D, Kruse RL, Vogel TR et al. R et al., 2022 [16]	HbA1c	Patients with higher HbA1c < 6.5% were at a significantly higher risk of 30-day readmission post-procedure (OR 1.06, 95% CI: 1.00–1.12, *p* = 0.04) compared to those with lower HbA1c < 6.5%.
Nativel M, Schneider F, Saulnier P-J, Gand E et al., 2018 [17]	HbA1c, tumor necrosis factor-α receptor 1 (TNFR1), angiopoietin-like 2, ischemia-modified albumin (IMA), CRP	Between the third and first tertiles, TNFR1 (HR 2.16, 95% CI: 1.19–3.92, *p* = 0.01), CRP (HR 7.14, 95% CI: 1.82–27.96; *p* = 0.005), and IMA (HR 2.04, 95% CI: 1.17–3.57, *p* = 0.01) were significantly associated with incidence of LEAD after adjusting for covariates.
Nativel M., Schneider F., Saulnier P., Meilhac O., Rondeau P., Cournot M. et al., 2018 [18]	HbA1c, tumor necrosis factor-α receptor 1 (TNFR1), angiopoietin-like 2, ischemia-modified albumin (IMA), CRP	Between the third and first tertiles, TNFR1 (HR 2.16, 95% CI: 1.19–3.92, *p* = 0.01) was significantly associated with a need for revascularization. Between the third and first tertile, CRP was significantly associated with LEAD (HR 7.14, 95% CI: 1.82–28.0; *p* = 0.005).
Martinez-Aguilar E, MD P, Orbe J, Fernandez-Montero A, Fernandez-Alonso S, Rodriguez J et al., 2017 [19]	High-density lipoprotein (HDL)	Patients with normal levels of HDL had a reduced incidence of mortality (HR 0.34, 95% CI: 0.21–0.57)
Mueller T., Dieplinger B., Forstner T., Poelz W., Haltmayer M., 2010 [20]	Pregnancy-associated plasma protein-A (PAPP-A)	PAPP-A was significantly associated with 5-year mortality (RR 1.31, 95% CI: 1.01–1.73, *p* = 0.024)
Gremmel T, Koppensteiner R, Ay C, Panzer S., 2014 [21]	Thrombin, and P-Selectin	<390 nM was established as a cut-off for peak thrombin to predict atherothrombotic events with a sensitivity of 85.7% and a specificity of 67%. P-selectin was elevated in patients with future atherothrombotic events but non-significantly (*p* = 0.08)
Skoglund PH, Arpegard J, Ostergren J, Svensson P., 2014 [22]	Amino-terminal pro-B-type natriuretic peptide (NT-proBNP), high-sensitivity C-reactive protein (hs-CRP), and cystatin.	Log(NT-proBNP) (HR 1.68, 95% CI: 1.09–2.60, *p* < 0.05) and Log(hs-CRP) (HR 1.53, 95% CI: 1.13–2.08, *p* < 0.01) were significantly associated with cardiovascular events. There were no associated between cystatin and cardiovascular events.
Sobel M, Yagi M, Moreno K, Kohler TR, Tang GL, Wijelath ES et al., 2019 [23]	Anti-phosphorylcholine IgM, CRP, IL-6	Patients in the lowest quartile of re-operative anti-PC IgM were at significantly higher risk of graft failure (*p* = 0.03, HR 2.11, 95% CI 1.09–4.07). IL6 and CRP were not associated with graft failure.
Skau E., Wagner P., Leppert J., Arnlov J., Hedberg P., 2023 [24]	N-terminal pro b-type natriuretic peptide (NT-proBNP) and Growth differentiation factor 15 (GDF-15)	NT-proBNP (HR: 1.59 95% CI: 1.32–1.91, *p* < 0.001) and GDF-15 (HR 2.32, 95% CI: 1.70–3.17, *p* < 0.001) were associated with 5-year adverse cardiovascular outcomes.
Stone PA, Thompson SN, Williams D, AbuRahma Z, Grome L, Schlarb H et al., 2015 [25]	Highly sensitive C-reactive protein (hs-CRP) and Brain natriuretic peptide (BNP)	Elevated BNP levels were associated with CV events (HR 3.5, 95% CI: 1.2–10.3, *p* = 0.03). CRP levels were not associated with cardiovascular events
Stone PA, Schlarb H, Campbell JE, Williams D, Thompson SN, John M et al., 2014 [26]	Highly sensitive C-reactive protein (hs-CRP) and Brain natriuretic peptide (BNP)	Elevated pre-operative hsCRP was associated with major adverse limb events, and elevated BNP and hsCRP were associated with CV events. Patients with higher baseline hsCRP and BNP experienced a 10.6-fold increase in major adverse cardiovascular events (95% CI: 2.6–42.9, *p* = 0.001).
Bleda S, de Haro J, Varela C, Acin F., 2015 [27]	C-reactive protein (CRP)	Reintervention within 1-year post-EVT was associated with baseline CRP levels (HR 1.1, 95% CI: 1.05–1.2, *p* < 0.001).
Shahin Y, Hatfield J, Chetter I, MD F., 2012 [28]	C-reactive protein (CRP)	High CRP at intermittent claudication diagnosis increased the 10-year risk of CV events (21% ± 11%; correlation coefficient with FRS and log-CRP r = 0.34, *p* = 0.002).
van Wijk DF, Boekholdt SM, Wareham NJ, Ahmadi-Abhari S, Kastelein JJP, Stroes ESG et al., 2013 [29]	C-reactive protein (CRP)	High CRP predicted non-fatal PAD events (HR 1.36, 95% CI: 1.26–1.48, *p* < 0.001). The fourth quartile of CRP levels had a significantly increased risk of non-fatal PAD events (HR 2.48, 95% CI: 1.85–3.32, *p* < 0.001).
Di X, Han W, Zhang R, Liu C, Zheng Y., 2022 [30]	C-reactive protein (CRP)	High hsCRP (HR 4.015, 95% CI, 1.628–10.551, *p* = 0.003) was associated with major adverse limb events.
Vrsalovic M, Vucur K, Car B, Krcmar T, Vrsalovic Presecki A., 2015 [31]	C-reactive protein (CRP)	High CRP independently predicted major adverse cardiovascular events. Patients with high CRP (along with renal dysfunction) at baseline had a 3.59-fold increase in major adverse cardiovascular event risk than controls (95% CI = 1.89–6.83, *p* < 0.001).
McDermott MM, Liu K, Green D, Greenland P, Tian L, Kibbe M et al., 2015 [32]	D-dimer, C-reactive protein (CRP), and serum amyloid A (SAA)	D-dimer levels were higher 8 months (*p* = 0.028), 6 months (*p* = 0.005), and 4 months (*p* = 0.017) before any ischemic heart disease (IHD) events (myocardial infarctions, IHD death, or unstable angina). CRP and SAA levels were not different between time periods leading up to IHD event.
Takamura T-A, Tsuchiya T, Oda M, Watanabe M, Saito R, Sato-Ishida R et al., 2017 [33]	Malondialdehyde-modified low-density lipoprotein (MDA/LDL), IL-6; high-sensitivity C-reactive protein (hsCRP), and D-dimer	MDA/LDL ratios (comparing post- and pre-EVT values) divided participants into high (≥0.495) and low (<0.495) cohorts. The low-ratio cohort had more limb-related events or death (*p* < 0.001) and clinical endpoints (HR 0.4210, *p* = 0.0154). Lower MDA/LDL and pre-EVT hsCRP were found to have an inverse relationship (r = −0.42, *p* = 0.012). D-dimer (*p* < 0.01) and IL-6 did increase post-EVT (<0.001) but were not associated with adverse clinic outcomes.
Wu S., Hsu L.-A., Cheng S.-T., Teng M.-S., Yeh C.-H., Sun Y.-C. et al., 2014 [34]	YKL-40 (cytokine)	YKL-40 level was associated with the PAD risk (*p* = 3.3 × 10^−23^).
Yang Y, Zhao X, Tang X, Lu J, Zhou M, Wang W et al., 2014 [35]	Serum cystatin C (Cys C), creatinine (sCr), pre-albumin	When sCr increased by ≥25%, there was no association with major adverse events. When Cys C increased by ≥5%, there was an association with major adverse events (HR: 3.576, 95% CI: 1.354–9.447, *p* = 0.010). Low prealbumin levels (HR: 0.000, 95% CI: 0.000–0.0269, *p* = 0.022) and high serum Cys C predicted 1-year major adverse events.
Li B., Nassereldine R., Zamzam A., Syed M.H., Mamdani M., Al-Omran M. et al., 2024 [36]	N-terminal pro-B-type natriuretic peptide [NT-proBNP], fatty acid binding protein 3 [FABP3], and FABP4	Three-year major adverse limb event predictors include (1) FABP, (2) FABP4, and (3) NT-proBNP. The prediction model created had an area under the ROC = 0.88 (95% CI, 0.84–0.94); sensitivity, 88%; specificity, 84%; PPV, 83%; and NPV, 91%.
Al-Thani H, El-Matbouly M, Al-Sulaiti M, Al-Thani N, Asim M, El-Menyar A., 2019 [37]	Hemoglobin A1c (HbA1c)	Despite having poorer glycemic control, patients with perioperative HbA1c values between 8.5 and 9.4% [HR 0.57 (95% CI 0.35–0.93)] and ≥9.5% [HR 0.46 (95% CI 0.31–0.69)] had a lower risk of morality after a lower extremity amputation compared to patients with perioperative HbA1c values < 8.5%, although they were not statistically significant after adjustments for age and sex.
Li B, Zamzam A, Syed M, Djahanpour N, Jain S, Abdin R et al., 2023 [38]	Fatty acid binding protein 4 [FABP4]	Each one-unit increase in FABP4 was significantly associated with major adverse limb events (unadjusted HR 1.19, 95% CI 1.04–1.27; adjusted HR 1.18, 95% CI 1.03–1.27; *p* = 0.022) and worsening PAD (unadjusted HR 1.18, 95% CI 1.13–1.31; adjusted HR 1.17, 95% CI 1.12–1.28; *p* < 0.001).
Ding N, Yang C, Ballew S, Kalbaugh C, McEvoy J, Salameh M et al., 2020 [39]	High-sensitivity C-reactive protein (hs-CRP), Galectin-3	After adjusting for risk factors, higher log-galectin-3 levels independently increased PAD and CLI risk, with hazard ratios of 1.17 (95% CI, 1.05–1.31) for PAD and 1.25 (95% CI, 1.05–1.49) for CLI per standard deviation. The highest quartile of galectin-3 also showed significantly higher risks for PAD (HR = 1.52) and CLI (HR = 1.85). Higher hs-CRP levels were also associated with more than twice the risk of PAD and CLI, with individuals in the highest quartile having adjusted hazard ratios (HR) of 2.19 (95% CI, 1.50–3.22) for PAD and 2.22 (95% CI, 1.15–4.29) for CLI.
Hjellestad ID, Softeland E, Husebye ES, Jonung T., 2018 [40]	Hemoglobin A1c (HbA1c)	HbA1c was significantly associated with all-cause mortality [HR: 1.75, 95% CI 1.24–2.46, *p* = 0.01] even after adjustments for age, sex, platelet inhibitors, statins, and antihypertensive medications [HR: 1.54, 95% CI 1.03–2.32, *p* = 0.04], although it was no longer significant after a fully adjusted analysis [HR: 1.39, 95% CI 0.92–2.309, *p* = 0.13].
Arya S, Binney ZO, Khakharia A, Long CA, Brewster LP, Wilson PW et al., 2018 [41]	Hemoglobin A1c (HbA1c)	Elevated HbA1c levels were associated with progressively higher risks of amputation: 26% (HR 1.26, 95% CI 1.15–1.39) for levels of 6.1–7.0%, 53% (HR 1.53, 95% CI 1.37–1.70) for levels of 7.1–8.0%, and 105% (HR 2.05, 95% CI 1.87–2.26) for levels above 8%, compared to those with HbA1c ≤ 6.0%. Similarly, the risk of major adverse limb events increased by 5% (HR 1.05, 95% CI 0.99–1.11), 21% (HR 1.21, 95% CI 1.13–1.29), and 33% (HR 1.33, 95% CI 1.25–1.42) for the respective HbA1c levels compared to those with HbA1c ≤ 6.0%.
Pohlhammer J, Kronenberg F, Rantner B, Stadler M, Peric S, Hammerer-Lercher A et al., 2014 [42]	High-sensitivity cardiac troponin T (hs-cTnT)	In PAD patients, detectable hs-cTnT predicted prevalent CVD (OR = 3.42, 95% CI, 1.68–7.00, *p* = 0.001) and was a strong predictor of all-cause mortality, especially at levels ≥ 14 ng/L (HR = 5.06, 95% CI, 2.12–12.17, *p* < 0.001). Levels ≥ 14 ng/L were also linked to incident CVD (HR = 3.15, 95% CI, 1.26–7.89, *p* = 0.01).
Chahrour MA, Kharroubi H, Al Tannir AH, Assi S, Habib JR, Hoballah JJ., 2021 [43]	Albumin	Serum albumin < 2.5 g/dL was strongly associated with increased mortality (adjusted odds ratio [AOR] = 2.25, 95% CI, 1.97–2.56, *p* < 0.001) and unplanned reoperation (AOR = 1.37, 95% CI, 1.26–1.49, *p* < 0.001) compared to normal albumin levels. Albumin levels between 2.5 and 3.39 g/dL also independently predicted higher mortality, with <2.5 g/dL levels showing the greatest risk.
Westfall JC, Cheng TW, Farber A, Jones DW, Eslami MH, Kalish JA et al. A et al., 2019 [44]	Albumin	Severe hypoalbuminemia was significantly associated with higher rates of 30-day readmission (40% vs. 30.8% vs. 17.8%, *p* = 0.005), 90-day ER visits (55.6% vs. 33.8% vs. 29.6%, *p* = 0.006), and 90-day readmission (66.7% vs. 48.9% vs. 35.6%, *p* = 0.001) rates compared to moderate hypoalbuminemia and normal albumin levels. Severe hypoalbuminemia was independently associated with a greater likelihood of 90-day ER visits (OR = 2.8, 95% CI, 1.23–6.36, *p* = 0.014) and 90-day readmission (OR = 2.63, 95% CI, 1.21–5.71, *p* = 0.015).
Peacock MR, Farber A, Eslami MH, Kalish JA, Rybin D, Doros G et al., 2017 [45]	Albumin	Serum album < 2.8 g/dL demonstrated significantly higher risk of 30-day mortality (OR 2.5, 95% CI 1.6–3.8, *p* < 0.001), return to the operating room in 30 days (OR 1.6, 95% CI 1.3–2.0, *p* ≤ 0.001), and prolonged length of stay (adjusted means ratio 1.2, 95% 1.1–1.2, *p* ≤ 0.001) when compared to individuals with serum album ≥ 3.5 g/dL.
Urbonaviciene G, Frystyk J, Urbonavicius S, Lindholt JS., 2014 [46]	Insulin-like growth factor-I (IGF-I) and insulin-like growth-factor-binding protein 2 (IGFBP-2).	An increase of 100 μg/L in baseline IGFBP-2 was significantly associated with a higher risk of CVD mortality [adjusted HR 1.12, 95% CI: 1.01–1.24]. The receiver-operating curve showed a significant area under the curve of 0.61 (95% CI: 0.51–0.67, *p* = 0.022), demonstrating that the model has limited ability to predict CVD mortality risk using IGFBP-2. IGF-I demonstrated insignificant positive associations with all-cause [HR 1.06, 95% CI 0.74–1.54] and CVD mortality [HR 1.18, 95% CI 0.7–1.99].
Ishii H, Kumada Y, Takahashi H, Toriyama T, Aoyama T, Tanaka M et al., 2012 [47]	Hemoglobin A1c (HbA1c)	The incidence of PAD (HR 1.63, 95% CI 1.17, 2.28, p0.0038) and limb amputation (HR 2.99, 95% CI 1.17, 7.70, p0.023) significantly increased in diabetic patients with HbA1c levels > 51 mmol/mol compared to those with ≤51 mmol/mol.
Wachsmann A, Maga M, Schonborn M, Olszewska M, Blukacz M, Cebenko M et al., 2021 [48]	Hemoglobin A1c (HbA1c)	Patients with HbA1c levels > 8.0% had significantly more restenosis (35.48% vs. 9.09%, *p* = 0.03), reduced Ulcer healing at 6 months (16.13% vs. 45.0%, *p* = 0.02), compared to patients with HbA1c levels ≤ 8%. No significant differences were found in 12-month major adverse cardiovascular events, death, or amputation rates between groups. Patients with HbA1c ≤ 8.0% showed better quality of life improvements in activities, symptoms, emotional well-being, and social well-being.
Gremmel T, Steiner S, Seidinger D, Koppensteiner R, Panzer S, Kopp CW., 2014 [49]	GPIIb/IIIa, TRAP-6, P-selectin	Levels of TRAP-6 inducible GP11b/IIIa > 3.23 MFI were associated with a 2.9-fold increased risk of non-fatal myocardial infarction, ischemic stroke or transient ischemic attack, and recurrent PAD symptoms (95% CI: 1.1–7.5; *p* = 0.04). Further, TRAP-6 inducible P-selectin levels above 40.2 MFI were significantly associated with a 3-fold-increased risk of these outcomes (95% CI: 1.3–7; *p* = 0.009).
Biscetti F, Ferraro P, Hiatt W, Angelini F, Nardella E, Cecchini A et al., 2019 [50]	Osteoprotegerin (OPG), tumor necrosis factor-α (TNF-α), interleukin-6 (IL-6), and C-reactive protein (CRP).	OPG, TNF-α, IL-6, and CRP all exhibit significant correlation to risk of MALE at 12 months after baseline (*p* < 0.001) as well as exhibiting a linear association to risk to MACE at 12 months after baseline (*p* < 0.001).
Cha J-J, Kim H, Ko Y-G, Choi D, Lee J-H, Yoon C-H et al., 2020 [51]	hemoglobin A1c (HbA1c)	The suboptimal glycemic control group (HbA1c ≥ 7.0) exhibited a higher incidence of MALEs in comparison to the optimal glycemic group (HbA1c < 7.0) (*p* = 0.072).
Saenz-Pipaon G., Ravassa S., Larsen K.L., Martinez-Aguilar E., Orbe J., Rodriguez J.A. et al., 2022 [52]	Lipocalin-2 (LCN2) and calprotectin	LCN2 and calprotectin increased the risk of cardiovascular-related death or amputation by 5.6 folds (*p* < 0.001) and 1.8 folds (*p* = 0.034), respectively.
Aday A,, Lawler P, Cook N, Ridker P et al., 2018 [53]	LDL particle (LDL-P), Tryglyceride-rich proteins, high-density lipoprotein (HDL)	High levels of total and small LDL particles were found to be associated with increased PAD risk (2.03; 95% CI, 1.14–3.59) (2.17; 95% CI, 1.10–4.27), whereas HDL particles were found to have an inverse relationship to PAD risk (0.29; 95% CI, 0.16 to 0.52; P trend < 0.0001).
Oshin OA, Torella F., 2013 [54]	Hemoglobin (Hb)	Twenty-six patients of the cohort experienced a MACE, showing a correlation between a decrease in hemoglobin below the mean and the MACE (1.4 [95% 1.13–1.7]; *p* = 0.002)
Urbonaviciene G, Frystyk J, Flyvbjerg A, Urbonavicius S, Henneberg EW, Lindholt JS., 2012 [55]	Plasma α-defensin and serum high sensitivity C reactive protein (hs-CRP)	High levels of α-defensin showed an increased risk of cardiovascular mortality (HR 3.04 95% CI 1.26–7.32; *p* = 0.013); hs-CRP was found to only be significantly correlated with cardiovascular mortality in univariate models but statistically insignificant in others (0.60, 95% CI 0.48–0.72; *p* = 0.089).
Martinez-Aguilar E, Gomez-Rodriguez V, Orbe J, Rodriguez J, Fernandez-Alonso L, MD P et al., 2015 [56]	Matrix metalloproteinase-10 (MMP-10), tissue inhibitor of matrix metalloproteinase 1 (TIMP-1)	MMP-10 was found in higher levels in PAD patients that underwent CLI (1086 ± 478 pg/mL vs. 822 ± 436 pg/mL; *p* < 0.001); univariate analysis found MMP-10 was increased with all-cause mortality and CV mortality (*p* < 0.03).
Amrock SM, Weitzman M., 2016 [57]	High-sensitivity CRP, Homocysteine	Homocysteine was found to be strongly correlated to all-cause mortality (HR 1.31, 95% CI: 1.11–1.54, *p* < 0.001).
Hobaus C, Herz CT, Wrba T, Koppensteiner R, Schernthaner G-H., 2020 [58]	HbA1C, C-reactive protein (CRP)	HbA1c > 7% (HR 1.3, 95% CI: 1.04–1.63) and elevated CRP (HR 1.5, 95% CI 1.2–2.0) were significantly associated with mortality.
Zhou Y, Zhang J, Zhu M, Lu R, Wang Y, Ni Z., 2015 [59]	Plasma Pentraxin 3 (PTX3)	Increased PTX3 exhibited a significantly poor outcome (*p* < 0.0001) and was further confirmed to be an independent predictor of overall mortality (HR, 1.105, 95% CI *p* = 0.03).
McGinigle KL, Kindell DG, Strassle PD, Crowner JR, Pascarella L, Farber MA et al. A et al., 2020 [60]	HbA1c	A significant correlation was found between hBA1c and MALEs, with very severe diabetes having 1.31 (95% CI, 1.10–1.55) times the odds of MALEs and 1.24 (95% CI, 0.82–1.87) times the odds of major limb amputation.
Abbas AE, Goodman LM, Timmis R, Boura J., 2010 [61]	Creatinine, hemoglobin	Increased preintervention creatinine (*p* = 0.015) and decreased preprocedural hemoglobin (*p* = 0.0068) were found to have correlation with endovascular intervention and target vessel revascularization. Preintervention creatinine was also found to be a significant predictor of mortality (*p* < 0.0001).
Guo S, Zhang Z, Jing Z et al., 2020 [62]	Interleukin-6 (IL-6)	Elevated Interleukin-6 levels at baseline (OR 1.11, 95% CI: 1.00–1.23, *p* = 0.044) were associated with 6-month in-stent stenosis.
Lee A, Haddad D, Rybin D, Howell C, Ghaderi I, Berman S et al., 2021 [63]	HbA1c	Elevated HbA1c levels were protective from death within 1 year for HbA1c > 6.5% versus <5.7% (HR 0.75, 95% CI: 0.61–0.93, *p* = 0.01.
Ding N, Kwak L, Ballew SH, Jaar B, Hoogeveen RC, Ballantyne CM et al., 2018 [64]	HbA1c	HbA1c > 7% was significantly associated with the risk of PAD (HR 6.00, 95% CI: 3.73–9.66) and CLI (HR 10.39, 95% CI: 4.79–22.53).
Ding N, Kwak L, Ballew SH, Jaar B, Hoogeveen RC, Ballantyne CM et al., 2018 [64]	Glycohemoglobin, Glycoalbumin	HbA1c > 7% was significantly associated with risk of PAD (HR 6.00, 95% CI: 3.73–9.66) and CLI (HR 10.39, 95% CI: 4.79–22.53).

## Data Availability

No new data were created or analyzed in this study. Data sharing is not applicable to this article.
